# Angiogenic Factor AGGF1 Activates Autophagy with an Essential Role in Therapeutic Angiogenesis for Heart Disease

**DOI:** 10.1371/journal.pbio.1002529

**Published:** 2016-08-11

**Authors:** Qiulun Lu, Yufeng Yao, Zhenkun Hu, Changqing Hu, Qixue Song, Jian Ye, Chengqi Xu, Annabel Z. Wang, Qiuyun Chen, Qing Kenneth Wang

**Affiliations:** 1 Key Laboratory of Molecular Biophysics of the Ministry of Education, College of Life Science and Technology and Center for Human Genome Research, Huazhong University of Science and Technology, Wuhan, P. R. China; 2 Duke University, Durham, North Carolina, United States of America; 3 Center for Cardiovascular Genetics, Department of Molecular Cardiology, Cleveland Clinic, Cleveland, Ohio, United States of America; 4 Department of Molecular Medicine, Cleveland Clinic Lerner College of Medicine, Case Western Reserve University, Cleveland, Ohio, United States of America; 5 Department of Genetics and Genome Sciences, Case Western Reserve University, Cleveland, Ohio, United States of America; University of Pittsburgh, UNITED STATES

## Abstract

AGGF1 is an angiogenic factor with therapeutic potential to treat coronary artery disease (CAD) and myocardial infarction (MI). However, the underlying mechanism for AGGF1-mediated therapeutic angiogenesis is unknown. Here, we show for the first time that AGGF1 activates autophagy, a housekeeping catabolic cellular process, in endothelial cells (ECs), HL1, H9C2, and vascular smooth muscle cells. Studies with *Atg5* small interfering RNA (siRNA) and the autophagy inhibitors bafilomycin A1 (Baf) and chloroquine demonstrate that autophagy is required for AGGF1-mediated EC proliferation, migration, capillary tube formation, and aortic ring-based angiogenesis. *Aggf1*^*+/-*^ knockout (KO) mice show reduced autophagy, which was associated with inhibition of angiogenesis, larger infarct areas, and contractile dysfunction after MI. Protein therapy with AGGF1 leads to robust recovery of myocardial function and contraction with increased survival, increased ejection fraction, reduction of infarct areas, and inhibition of cardiac apoptosis and fibrosis by promoting therapeutic angiogenesis in mice with MI. Inhibition of autophagy in mice by bafilomycin A1 or in *Becn1*^*+/-*^ and *Atg5* KO mice eliminates AGGF1-mediated angiogenesis and therapeutic actions, indicating that autophagy acts upstream of and is essential for angiogenesis. Mechanistically, AGGF1 initiates autophagy by activating JNK, which leads to activation of Vps34 lipid kinase and the assembly of Becn1-Vps34-Atg14 complex involved in the initiation of autophagy. Our data demonstrate that (1) autophagy is essential for effective therapeutic angiogenesis to treat CAD and MI; (2) AGGF1 is critical to induction of autophagy; and (3) AGGF1 is a novel agent for treatment of CAD and MI. Our data suggest that maintaining or increasing autophagy is a highly innovative strategy to robustly boost the efficacy of therapeutic angiogenesis.

## Abstract 2

Treatment with the angiogenic factor AGGF1 dramatically improves survival and cardiac function in mouse models for coronary artery disease and myocardial infarction by activating autophagy and angiogenesis.

## Author Summary

Coronary artery disease is the number one killer disease worldwide. Recently, therapeutic angiogenesis has been proposed as an attractive new strategy for treating this and other ischemic diseases. This study establishes the angiogenic factor AGGF1 as a novel target and agent that can successfully treat coronary artery disease and acute myocardial infarction and dramatically improve survival and cardiac function in mouse models. We present the unexpected finding that AGGF1 has these effects via activating autophagy, and that autophagy is essential for therapeutic angiogenesis in animals. We find that AGGF1 is a novel master regulator of autophagy not only in endothelial cells but also in all other cell types examined in the study. Mechanistically, AGGF1 activates autophagy by activating JNK, which leads to activation of the Vps34 lipid kinase and assembly of the Becn1-Vps34-Atg14 complex involved in the initiation of autophagy. The study thus provides a link connecting the therapeutic angiogenesis and autophagy pathways in heart disease.

## Introduction

AGGF1 is an Angiogenic factor with a G-patch domain and a Forkhead-associated (FHA) domain. AGGF1 was initially identified by our laboratory through positional cloning analysis for a gene involved in development of Klippel–Trénaunay syndrome (KTS), a congenital vascular disorder [1]. AGGF1 can induce angiogenesis and excessive angiogenesis, and increased AGGF1 expression is a cause of KTS [1–5]. We and others have also found that AGGF1 is critical to specification of veins [6,7], specification of multipotent hemangioblasts [8], and anti-inflammation [9]. However, the molecular mechanisms underlying these processes remain to be fully defined.

Coronary artery disease (CAD) and its most severe manifestation, myocardial infarction (MI), are the most common causes of death worldwide. Therapeutic angiogenesis has been proposed as an attractive new strategy to treat CAD and MI patients. Therapeutic angiogenesis can be defined as the utilization of angiogenic growth factors to promote neovascularization and growth of collateral blood vessels, which act as endogenous bypass conduits to improve blood flow and increase tissue perfusion in the ischemic extremity. However, there is currently no United States Food and Drug Administration (FDA)-approved therapeutic angiogenesis to treat CAD, MI, or other ischemic diseases [10,11]. Many challenges must be overcome before therapeutic angiogenesis becomes an applied patient therapy, including the critical identification of the most robust, effective angiogenic factor [10,11]. Importantly, lack of understanding of the fundamental molecular mechanisms underlying therapeutic angiogenesis has slowed advances in this field.

Autophagy is an evolutionarily conserved dynamic catabolic process that removes damaged, dysfunctional organelles and long-lived protein aggregates [12]. It recycles amino acids and other substrates for protein synthesis and ATP generation [12]. However, excessive autophagy can also lead to cell death. Autophagy is initiated by the formation of the phagophore; this process is mediated by the class III PI3-K complex consisting of Vps34, Vps15, Atg14, and beclin 1 [12]. The phagophore then elongates and engulfs cytoplasmic materials targeted for degradation, leading to the formation of autophagosome. During this process, the microtubule-associated protein 1 light chain 3 (LC3) is cleaved and converted to LC3-I. LC3-1, the soluble form of LC3, is then activated and converted to LC3-II, which is the autophagic vesicle-associated form of LC3. The autophagosome is fused with the lysosome to form the autolysosome, which leads to degradation of the vesicle content by lysosomal hydrolases, recycling of the degraded products into amino acids and lipids, and generation of ATP [12,13].

Here, we demonstrate that AGGF1 induces autophagy in endothelial cells (ECs) and all other cells analyzed as well as in mice with acute MI using a series of integrative in vitro and in vivo approaches. We show that an angiogenic factor can induce autophagy and that autophagy acts upstream of angiogenesis and is essential for therapeutic angiogenesis. We also demonstrate the robust potential of recombinant AGGF1 and AGGF1-mediated autophagy in therapeutic angiogenesis to treat acute MI.

## Results

### AGGF1 Is a Universal Master Regulator of Autophagy

**Fig 1:**
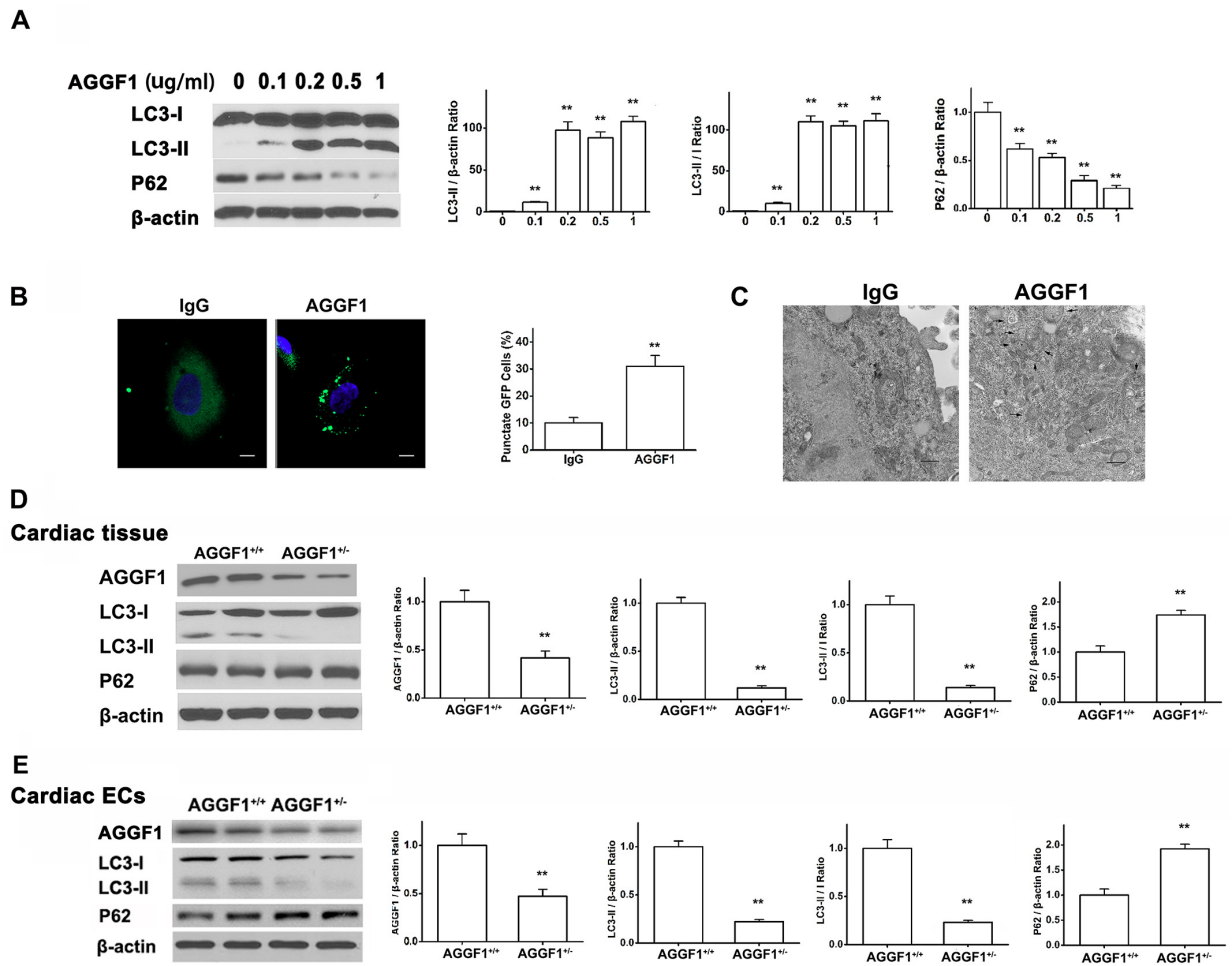
AGGF1 is involved with autophagy activity. **(A)** Western blot analysis showing that AGGF1 at different concentrations induces autophagy (increased LC3-II/β-actin and LC3-II/I; decreased p62 expression) in HUVECs under a normoxic condition for 4 h (*n* = 3/group). **(B)** AGGF1 (500 ng/ml) induces autophagy in HUVECs (representative images of LC3-GFP punctuate) (*n* = 6/group). Scale bar = 10 μm. **(C)** Representative electronic microscopic images of HUVECs treated with AGGF1 or control IgG showing detection of AGGF1-induced autophagy. Scale bar = 1 μm. **(D)** Western blot analyses for autophagy markers in cardiac tissues from *AGGF1*^*+/-*^ mice and wild-type *AGGF1*^*+/+*^ mice (*n* = 6/group). **(E)** Western blot analysis for autophagy markers in cardiac ECs isolated from *AGGF1*^*+/-*^ mice and wild-type *AGGF1*^*+/+*^ mice (*n* = 6/group). Underlying data are shown in S1 Data.

Because AGGF1 is an angiogenic factor, we focused our studies of AGGF1 on ECs. To identify the role of AGGF1 in regulating autophagy, we treated human umbilical vein endothelial cells (HUVECs) with different doses of AGGF1 and then analyzed expression levels of LC3-II and p62. AGGF1 strongly activated autophagy and the effect was concentration-dependent and saturable (Fig 1A). As a positive control, serum starvation induced the activation of autophagy (S1 Fig). To further demonstrate that AGGF1 induces autophagy, HUVECs were infected with a viral expression construct for Green Fluorescent Protein (GFP)-LC3 for 24 h and then treated with AGGF1 or control Immunoglobin G (IgG). HUVECs with GFP punctuate (autophagic cells with autophagic vesicle-bound LC3-II) and overall GFP-staining were counted (Fig 1B). The ratio of HUVECs with GFP punctuate was 31% ± 4% of the total GFP-positive cells with AGGF1 treatment but only 10% ± 2% for HUVECs incubated with IgG (Fig 1B). Electronic microscopy directly demonstrated that AGGF1 induced formation of autophagosome (Fig 1C). These data further support the finding that AGGF1 induces autophagy in ECs.

To determine whether AGGF1-activated autophagy is specific to ECs, we analyzed several other types of cells. As shown in S2 Fig, AGGF1 activated autophagy in all cells examined. These data suggest that AGGF1 is a universal master regulator of autophagy.

### AGGF1 Haploinsufficiency Inhibits Autophagy in the Heart

We created *Aggf1* knockout mice with a gene-trapping allele in intron 11. Homozygous *Aggf1*^*-/-*^ mice are embryonically lethal, but heterozygous *Aggf1*^*+/-*^ mice showed partial embryonic lethality, in which 60% of them can survive to adulthood. To further demonstrate the critical role of AGGF1 in autophagy, we isolated heart tissue samples from *Aggf1*^*+/-*^ mice and performed western blot analysis for autophagy markers LC3-II and p62. Compared to wild-type mice, *Aggf1*^*+/-*^ mice showed a significantly decreased expression level of LC3-II and a significantly increased expression level of p62 (Fig 1D). Moreover, cardiac ECs from wild-type or *Aggf1*^*+/-*^ KO mice showed reduced activation of autophagy compared with wild-type ECs (Fig 1E). These data indicate that reduced expression of AGGF1 by 50% inhibits autophagy, suggesting that AGGF1 is critically involved in autophagy.

Western blot analysis was performed with heart tissue lysates from 8-wk-old wild-type and *Aggf1*^*+/-*^ KO mice with apoptosis markers. *Aggf1*^*+/-*^ KO mice showed an increased level of poly (ADP-ribose) polymerase (PARP), caspase 3, or Bax, but a reduced level of Bcl-2 compared with wild-type mice (S3 Fig). No HIF1α was detected in either wild-type or *Aggf1*^*+/-*^ KO mice (S3 Fig). These data suggest that haploinsufficiency of *Aggf1* induced apoptosis of cardiomyocytes.

### AGGF1-Induced Autophagy Is Required for Angiogenesis

**Fig 2:**
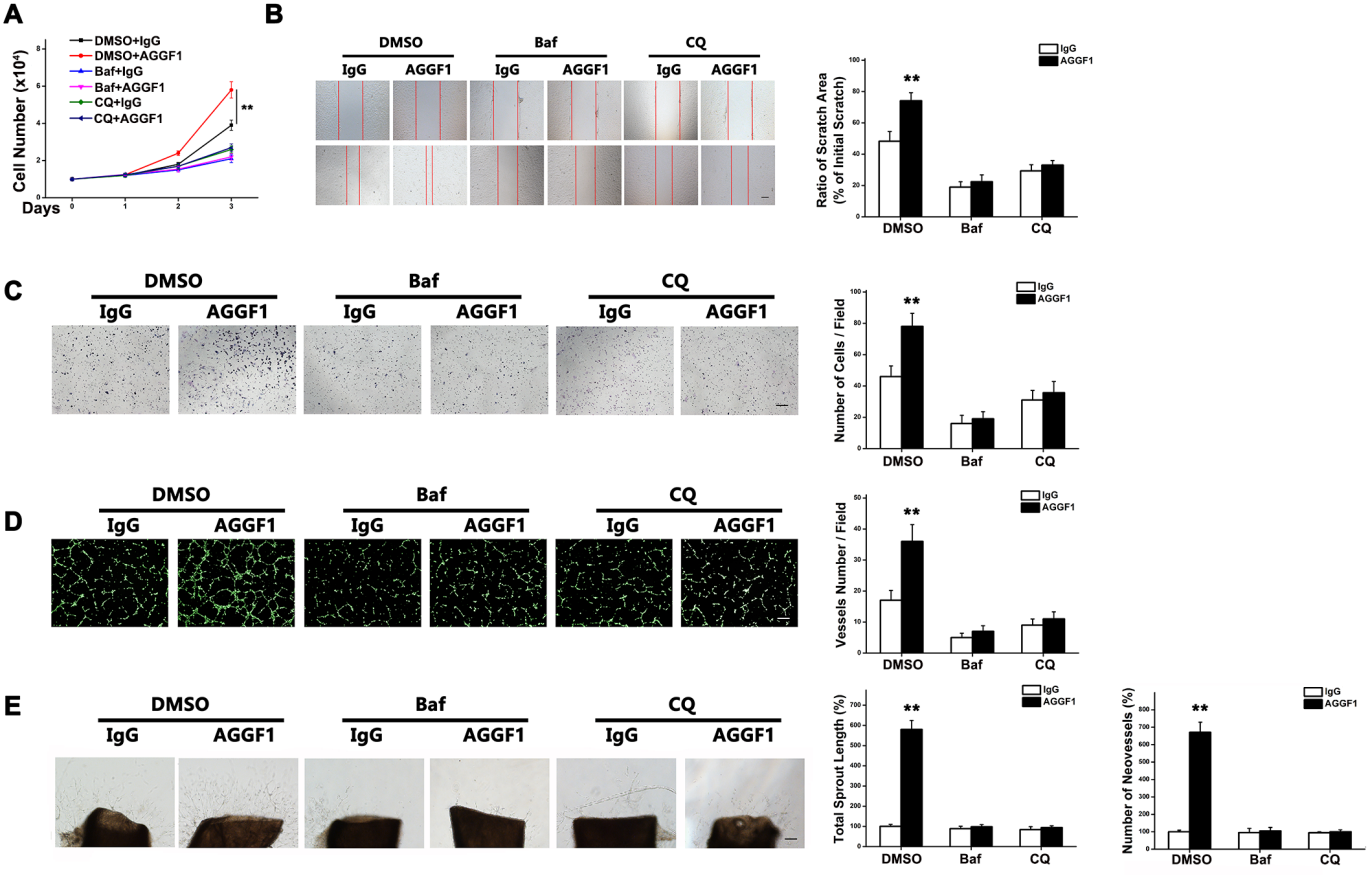
AGGF1-induced angiogenesis is suppressed by autophagic flux inhibition. **(A)** HUVEC proliferation induced by AGGF1 (500 ng/ml) was blocked by pretreatment with bafilomycin A1 (Baf) or chloroquine (CQ) for 2 h before treatment with IgG (control) or AGGF1 (*n* = 4/group). **(B)** Baf or CQ inhibited AGGF1-induced migration of HUVECs using scratch assays (*n* = 4/group). **(C)** Baf or CQ inhibited AGGF1-induced migration of HUVECs using Transwell migration assays (*n* = 4/group). **(D)** Baf or CQ inhibited AGGF1-induced endothelial tube formation (*n* = 4/group). Scale bar = 50 μm. **(E)** Baf or CQ inhibited AGGF1-induced sprout angiogenesis in an e*x vivo* aortic ring angiogenesis assay (*n* = 4/group). Scale bar = 125 μm. Underlying data are shown in S1 Data.

To distinguish the cause–effect relationship between AGGF1-promoted autophagy and AGGF1-induced angiogenesis, we performed a series of studies with two inhibitors of autophagy, bafilomycin A1 (Baf) and chloroquine (CQ), and DMSO control. The effectiveness of Baf and CQ on inhibition of autophagy was verified by western blot analysis (S4 Fig). Recombinant AGGF1 promoted HUVEC proliferation (compare DMSO+IgG and DMSO+AGGF1, Fig 2A). However, inhibition of autophagy by Baf or CQ completely blocked AGGF1-induced HUVEC proliferation (Fig 2A). These data suggest that autophagy is required for AGGF1-mediated EC proliferation.

EC migration is also one of the key processes involved in angiogenesis. Wound-healing scratch assays for migration showed that AGGF1 treatment significantly induced HUVEC migration, but this effect was blocked by Baf or CQ (Fig 2B). Similarly, with Transwell migration assays, AGGF1 in the bottom chamber induced migration of 1.64-fold more HUVECs to the bottom of Transwells than IgG. However, this effect was blocked by Baf or CQ (Fig 2C). These data suggest that autophagy is required for AGGF1-mediated EC migration.

We also assessed the effect of autophagy on capillary tube formation mediated by AGGF1 using a matrigel-based angiogenesis assay. AGGF1 treatment increased the number of endothelial cell tubes, but this effect was blocked by Baf or CQ (Fig 2D). Moreover, using an *ex vivo* endothelial cell sprout assay from aortic rings, it is obvious that sprouts emerged from the aorta rings and grew outward after 4 d in culture with AGGF1 (500 ng/ml) (compare AGGF1 and IgG in the DMSO group, Fig 2E). However, treatments with Baf or CQ resulted in a significant decrease in sprout length and density by AGGF1 as compared to IgG (Fig 2E). These data suggest that autophagy is required for AGGF1-mediated angiogenesis.

**Fig 3:**
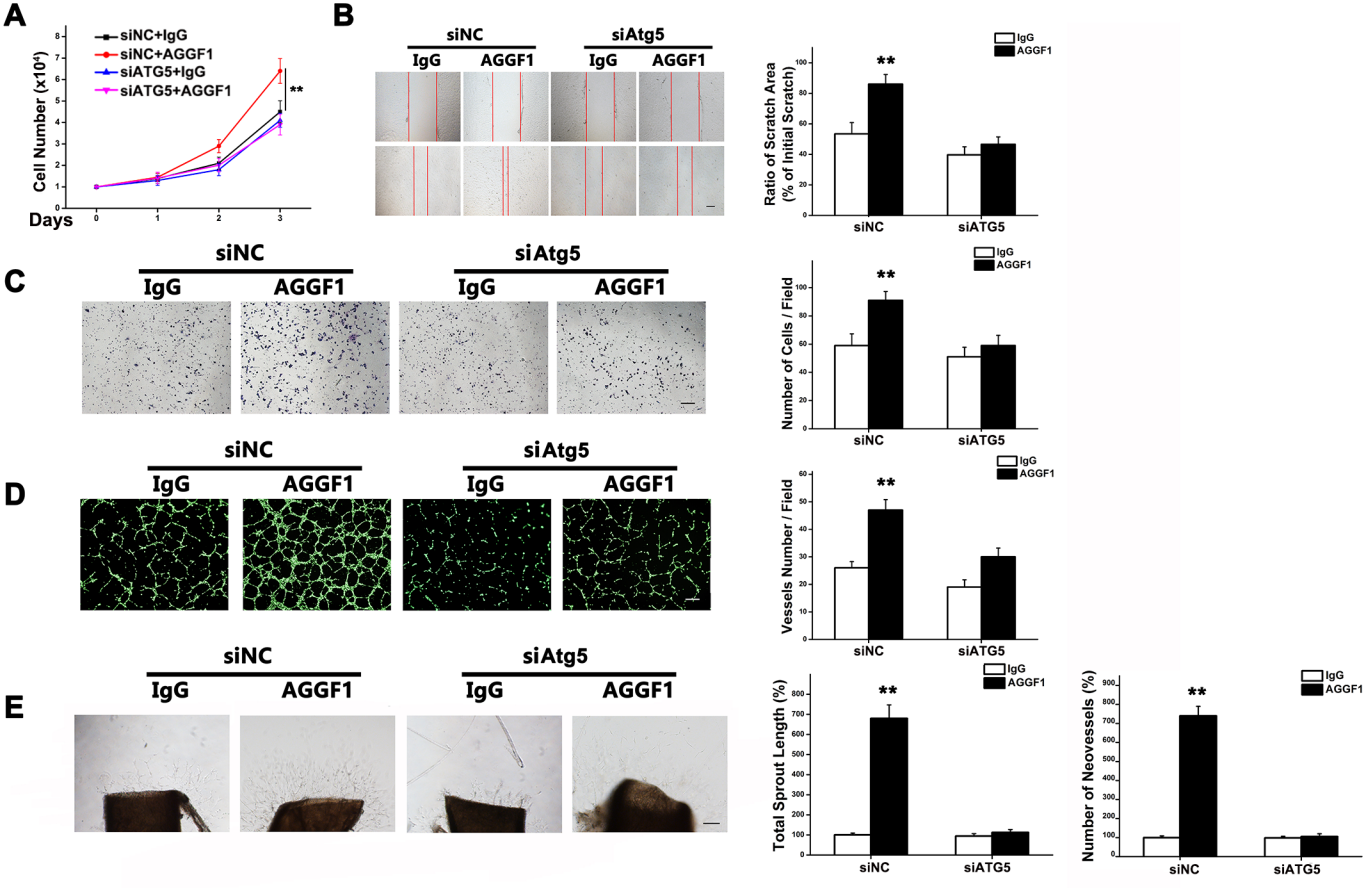
Atg5 knockdown prevents activation of angiogenesis by AGGF1. **(A)** HUVEC proliferation induced by AGGF1 (500 ng/ml) was blocked by *Atg5*-specific siRNA (*n* = 4/group). **(B)** *Atg5*-specific siRNA inhibited AGGF1-induced migration of HUVECs using scratch assays (*n* = 4/group). **(C)** *Atg5*-specific siRNA inhibited AGGF1-induced migration of HUVECs using Transwell migration assays (*n* = 4/group). **(D)** *Atg5*-specific siRNA inhibited AGGF1-induced endothelial tube formation (*n* = 4/group). Scale bar = 50 μm. **(E)** *Atg5*-specific siRNA inhibited AGGF1-induced sprout angiogenesis in an e*x vivo* aortic ring angiogenesis assay (*n* = 4/group). Scale bar = 125 μm. Underlying data are shown in S1 Data.

Similar to Baf or CQ, inhibition of autophagy by knockdown of Atg5 expression by siRNA (S5 Fig) also blocked EC proliferation (Fig 3A), wound-healing migration (Fig 3B), Transwell migration (Fig 3C), capillary tube formation (Fig 3D), and sprout angiogenesis from aortic rings (Fig 3E).

### AGGF1 Expression Is Increased after Acute MI

**Fig 4:**
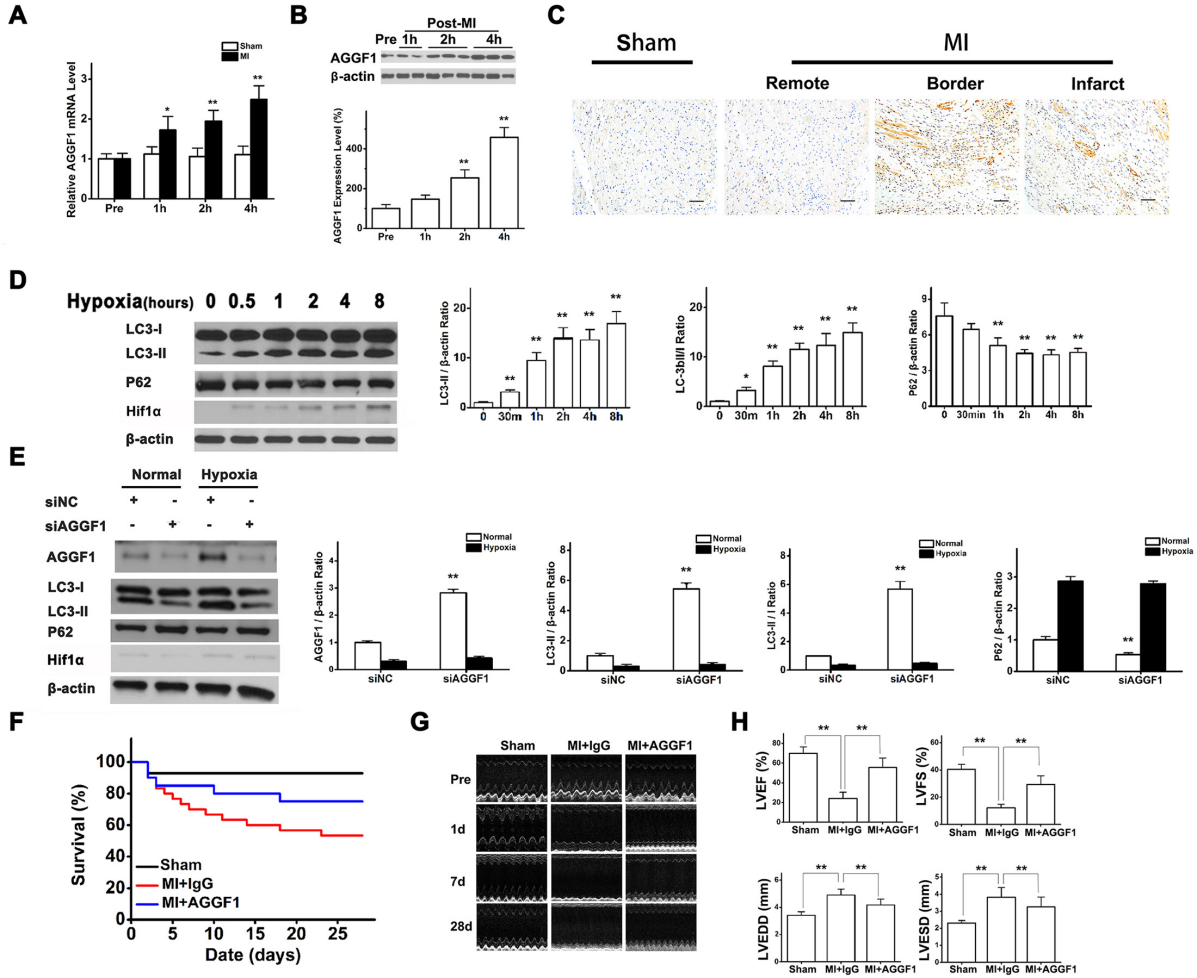
Up-regulation of AGGF1 in response to acute MI and robust therapeutic effects of AGGF1 protein therapy on MI. **(A)** Real-time reverse transcription (RT)-PCR analysis for *AGGF1* mRNA expression in mice with LAD ligation or sham operation (Sham) (*n* = 6/group). Pre, before surgery. **(B)** Western blot analysis for AGGF1 protein expression (*n* = 6 mice/group). **(C)** Immunostaining analysis for AGGF1 protein expression in cross-sections of hearts (*n* = 5). Scale bar = 50 μm. **(D)** AGGF1 (500 ng/ml) induces autophagy in HUVECs under a hypoxic condition for different time points (*n* = 3/group). **(E)** AGGF1-specific siRNA inhibited autophagy under normoxia or hypoxia. **(F)** Post-MI survival of mice with AGGF1 treatment (*n* = 20), mice with IgG treatment (*n* = 30), and sham mice (*n* = 14). **(G)** Representative M-mode echocardiograms. **(H)** Effects of AGGF1 protein therapy on myocardial function and contraction. Underlying data are shown in S1 Data.

We analyzed the expression level of *AGGF1* in response to acute MI in a mouse model with ligation of the left anterior descending (LAD) coronary artery. The expression levels of both the *AGGF1* mRNA and AGGF1 protein in heart tissue increased significantly in mice with MI compared to mice with sham operation (*p* < 0.05) (Fig 4A and 4B). Immunostaining with an anti-AGGF1 antibody showed that AGGF1 expression increased in the border zone and the infarct area of the left ventricle in mice with MI compared to the non-infarct area or similar areas in mice with sham operation (Fig 4C).

### Hypoxia Can Promote AGGF1 Expression in Endothelial Cells

To determine the mechanism by which MI induces *AGGF1* expression, we hypothesized that hypoxia after MI may be critical. To test the hypothesis, we examined the expression level of the AGGF1 protein in HUVECs in response to hypoxia. AGGF1 expression increased in HUVECs challenged with 1% oxygen for 30 min up to 8 h compared to cells cultured under a normal level of oxygen (S6A Fig). Similar findings were made for the *AGGF1* mRNA (S6B Fig). Western blot analysis showed that AGGF1 expression was increased in response to hypoxia in mouse cardiac ECs (S7 Fig). Together, these data suggest that hypoxia induces expression of *AGGF1*, providing a mechanism for ischemia-induced AGGF1 up-regulation in MI mice.

### AGGF1-Induced Autophagy under a Hypoxic Condition

In addition, the activity of AGGF1-promoted autophagy, i.e., the expression level of LC3-II or decreased expression of p62, increased as the culture time of HUVECs under hypoxia increased (Fig 4D). This may be related in part to the earlier finding that hypoxia induces increased AGGF1 expression (S6 Fig).

When *AGGF1* expression was knocked down in HUVECs with transfection of siRNA, hypoxia-induced autophagy was inhibited (Fig 4E). The data suggest that AGGF1 is critical to induction of autophagy under hypoxia.

### AGGF1 Protein Therapy Reduces Mortality and Dramatically Improves Cardiac Function after MI

To explore the functional effects of up-regulation of AGGF1 expression in response to MI, we tested the purified recombinant AGGF1 protein as a therapy for acute MI. The survival rate of MI mice with AGGF1 protein therapy was 80% 2 wk after treatment, a significant increase over the 60% rate for the group with IgG treatment (*p* < 0.01) (Fig 4F). Similarly, 4 wk after treatment, AGGF1 remarkably improved the post-MI survival rate to 75% from 53% for treatment with IgG (*p* < 0.01) (Fig 4F).

Echocardiography showed that AGGF1 dramatically improved cardiac functions after acute MI (Fig 4G). Left ventricular ejection fraction (LVEF) dramatically improved with AGGF1 treatment after MI. The significant improvement was obvious even at 14 d after AGGF1 treatment with LVEF of 50.29% ± 8.47% compared with 23.69% ± 7.49% with treatment of control IgG (*p* < 0.01; S8 Fig). At 28 d (end of study), LVEF more than doubled with AGGF1 treatment compared to control IgG treatment (55.57% ± 9.65% versus 22.77% ± 7.79%, *p* < 0.01) and reached almost the normal range of LVEF for mice (Fig 4H). Similar dramatic improvement was observed for LV fraction shortening (LVFS) with AGGF1 treatment compared to IgG treatment (29.42% ± 6.37% versus 14.35% ± 2.53% at 28 d after treatment, *p* < 0.01) (Fig 4H). Moreover, significant decreases were found with AGGF1 treatment compared to IgG treatment for the LV end-diastolic diameter (LVEDD) (4.17 ± 0.42 mm versus 4.89 ± 0.41 mm, *p* < 0.01) and LV end-systolic diameter (LVESD) (3.26 ± 0.58 mm versus 3.92 ± 0.57 mm, *p* < 0.01) (Fig 4H), indicating improvement of cardiac structure with AGGF1 protein therapy.

The new speckle tracking function of Vevo-2100 echocardiography can be used to quantify global and regional (e.g., infarct region) cardiac functions and estimate the size of infarct [14,15]. The peak longitudinal strain and strain ratios serve as markers for myocardial contractile states at earlier time points after MI and predict the later development of adverse LV remodeling (S9A Fig). Four weeks after treatment, the longitudinal strain of the global heart in the MI group with AGGF1 treatment was -8.03% ± 2.38%, a dramatic increase compared to that of IgG treatment (-2.85% ± 0.86%, *p* < 0.01) (S9B Fig). Identical results were observed for the longitudinal strain ratio of the global heart (-5.14% ± 1.56% for AGGF1 treatment versus -2.72% ± 0.74% for IgG treatment, *p* < 0.01) (S9C Fig and S1 Table). Myocardial contractile function in the regional infarct areas was also assessed. The longitudinal strain and longitudinal strain ratio in the infarct areas significantly improved with AGGF1 treatment compared to control IgG treatment (longitudinal strain: -4.39% ± 2.68% versus -1.625% ± 0.63%, *p* < 0.01; longitudinal strain ratio: -3.52% ± 1.54% versus -1.22% ± 0.11%, *p* < 0.01) (S9C Fig). These data suggest that AGGF1 dramatically improves the global cardiac function as well as myocardial contractile function in the regional infarct areas.

AGGF1 protein therapy inhibited cardiac hypertrophy after MI. At 28 d after treatment, AGGF1 treatment inhibited early cardiac hypertrophy associated with MI. As shown in S10 Fig, MI induced an increase of the ratio of heart weight (HW) to tibia length (TL) or body weight and the ratio of lung weight (LW) to TL or body weight, indicative of cardiac hypertrophy. However, AGGF1 protein therapy significantly attenuated MI-induced hypertrophy of the heart (S10 Fig).

### AGGF1 Induces Autophagy in Mice after MI

**Fig 5:**
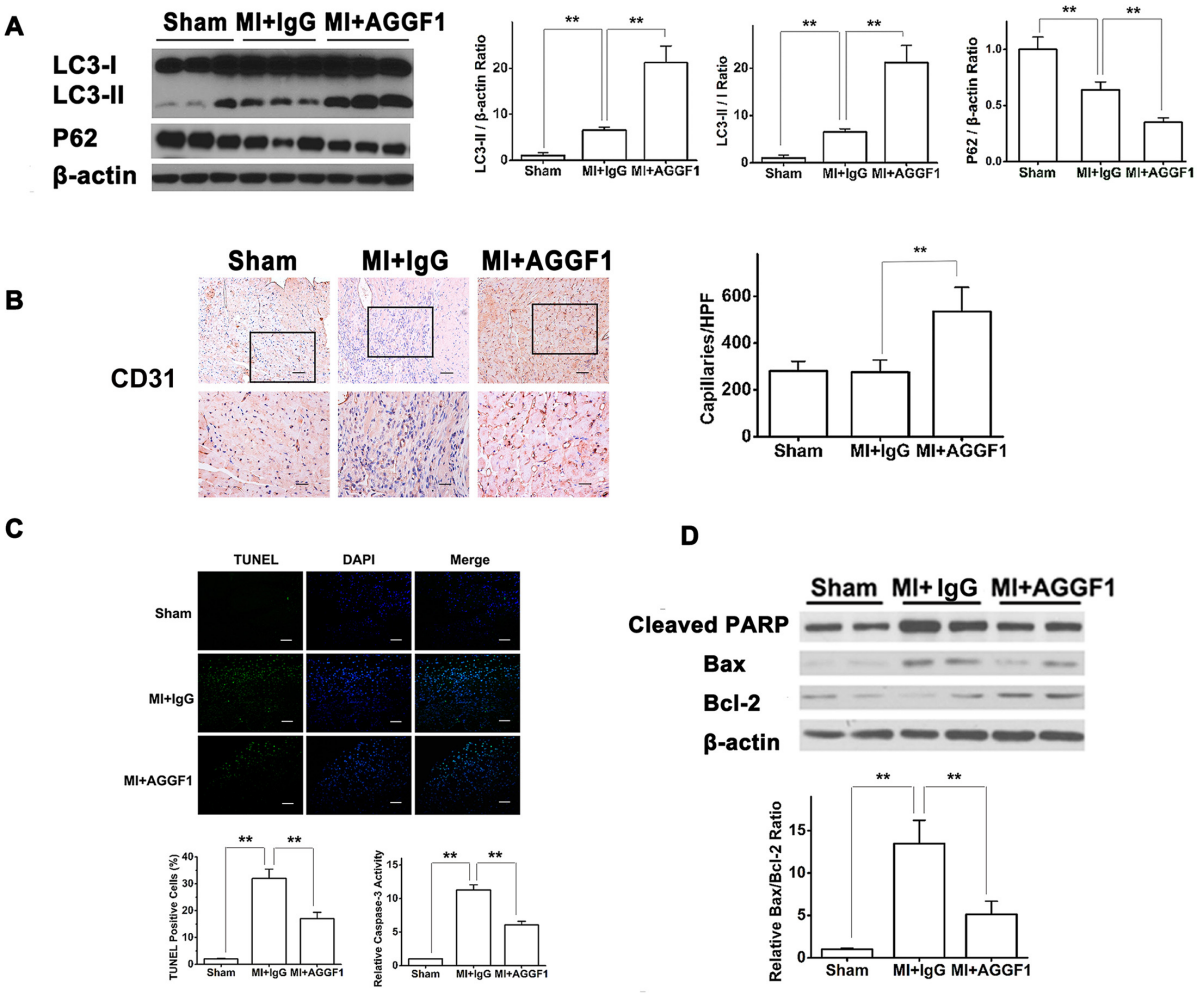
AGGF1 induces autophagy and angiogenesis and suppresses apoptosis in mice after MI. **(A)** Western blot analysis for LC3-II in cardiac tissues from mice with LAD ligation (*n* = 6/group). **(B)** CD-31 immunostaining. Densities of CD31-positive vessels are quantified and graphed (*n* = 5/group). HPF, high-power field. Scale bar = 50 μm and 25 μm. **(C)** TUNEL staining of cross-sections of mouse hearts. The number of TUNEL-positive myocytes in the infarct area is quantified and graphed (*n* = 5/group). Caspase-3 activity was measured in myocardial tissues (*n* = 5/group). Scale bar = 50 um. **(D)** Western blot analysis for cleaved PARP, Bax, and Bcl-2 from myocardial protein lysates (*n* = 3/group) and real-time RT-PCR for the ratio of *Bax/Bcl-2* mRNA levels (*n* = 3/group). Underlying data are shown in S1 Data.

To further confirm whether AGGF1 protein therapy reduces mortality and dramatically improves cardiac function after MI through autophagy, we determined the functional role of AGGF1 in autophagy in the infarcted heart. We found that, compared to sham-operated mice, mice with acute MI showed an increased expression level of LC3-II over control β-actin or LC3-I or decreased expression of p62 (Fig 5A). Surprisingly, the AGGF1-treated group showed a dramatic increase of LC3-II expression or decreased expression of p62 compared with the IgG-treated group (Fig 5A). These data indicate that AGGF1 induces autophagy in mice after MI in vivo.

### AGGF1 Promotes Angiogenesis after MI

To elucidate the mechanism by which AGGF1 protein therapy drastically improves myocardial function and contraction after acute MI, we examined whether AGGF1 promoted therapeutic angiogenesis. Immunostaining with an anti-CD31 antibody, an endothelial cell marker, was performed with cryo-sections from left ventricles. MI mice treated with AGGF1 showed much more CD31-positive capillaries than MI mice treated with control IgG (543 ± 143 versus 272 ± 72 capillaries/high-power field [HPF], *p* < 0.01) or sham mice (Fig 5B). We performed α-SMA staining for heart sections and showed that AGGF1 treatment increased the density of α-SMA-positive vessels (S11A Fig). These data suggest that AGGF1 promotes therapeutic angiogenesis after MI, resulting in improved myocardial contractile function.

### AGGF1 Protein Therapy Protects Hearts from Cell Death after MI

Cardiac apoptosis increases in response to MI and is responsible for myocardial fibrosis after MI. We assessed the cyto-protective activity of AGGF1 after MI by TUNEL staining of left ventricles. Administration of the recombinant AGGF1 protein enhanced myocardial cell survival by significantly decreasing the number of TUNEL-positive cells after MI (*p < 0*.*01)* (Fig 5C and S12 Fig). Twenty-eight days after AGGF1 treatment, myocardial tissue samples were collected and activity of caspase-3 was measured with myocardial lysates. AGGF1 protein reduced caspase-3 activity by 44.79% (*p < 0*.*01*) (Fig 5C).

Cardiac apoptosis is a major cause of cardiac fibrosis. H&E staining with Masson trichrome revealed that MI increased cardiac fibrosis in the left ventricle and reduced LV wall thickness (top panel, S13 Fig). AGGF1 protein therapy decreased MI-induced cardiac fibrosis and increased LV wall thickness (top panel, S13 Fig). The size of cardiac fibrotic areas reflecting the size of the infarct areas was increased by MI, but AGGF1 treatment dramatically reduced the infarct size (22.54% ± 1.25% for AGGF1 treatment versus 50.94% ± 7.74% for IgG treatment, *p* < 0.01) (bottom panel, S13 Fig). Moreover, AGGF1 treatment increased LV wall thickness compared to IgG treatment (510.47 ± 96.27 μm versus 266.89 ± 57.38 μm, *p* < 0.01) (bottom panel, S13 Fig). H&E staining showed that MI induced necrosis, but AGGF1 administration reduced the MI-induced necrosis (S11B Fig). These data are consistent with the echocardiographic data.

To further confirm that AGGF1 inhibited myocardial apoptosis after MI, western blot analysis with myocardial lysates was performed for cleaved PARP, Bax, and Bcl-2. MI increased the levels of cleaved PARP and Bax, indicating activation of Bax and PARP, but their activation was inhibited by AGGF1 protein therapy (Fig 5D). Consistent with the protective role of AGGF1, expression of anti-apoptotic protein Bcl-2 was increased by AGGF1 treatment (Fig 5D). Real-time RT-PCR analysis showed that the expression level of *Bcl-2* mRNA in myocardial tissue also increased in AGGF1-treated mice (data not shown). The ratio of *Bax/Bcl-2*, an indicator for apoptosis, decreased during AGGF1 protein therapy (Fig 5D).

### AGGF1 Haploinsufficiency Inhibits Autophagy, Decreases Survival, and Impairs Myocardial Function and Contraction after MI

**Fig 6:**
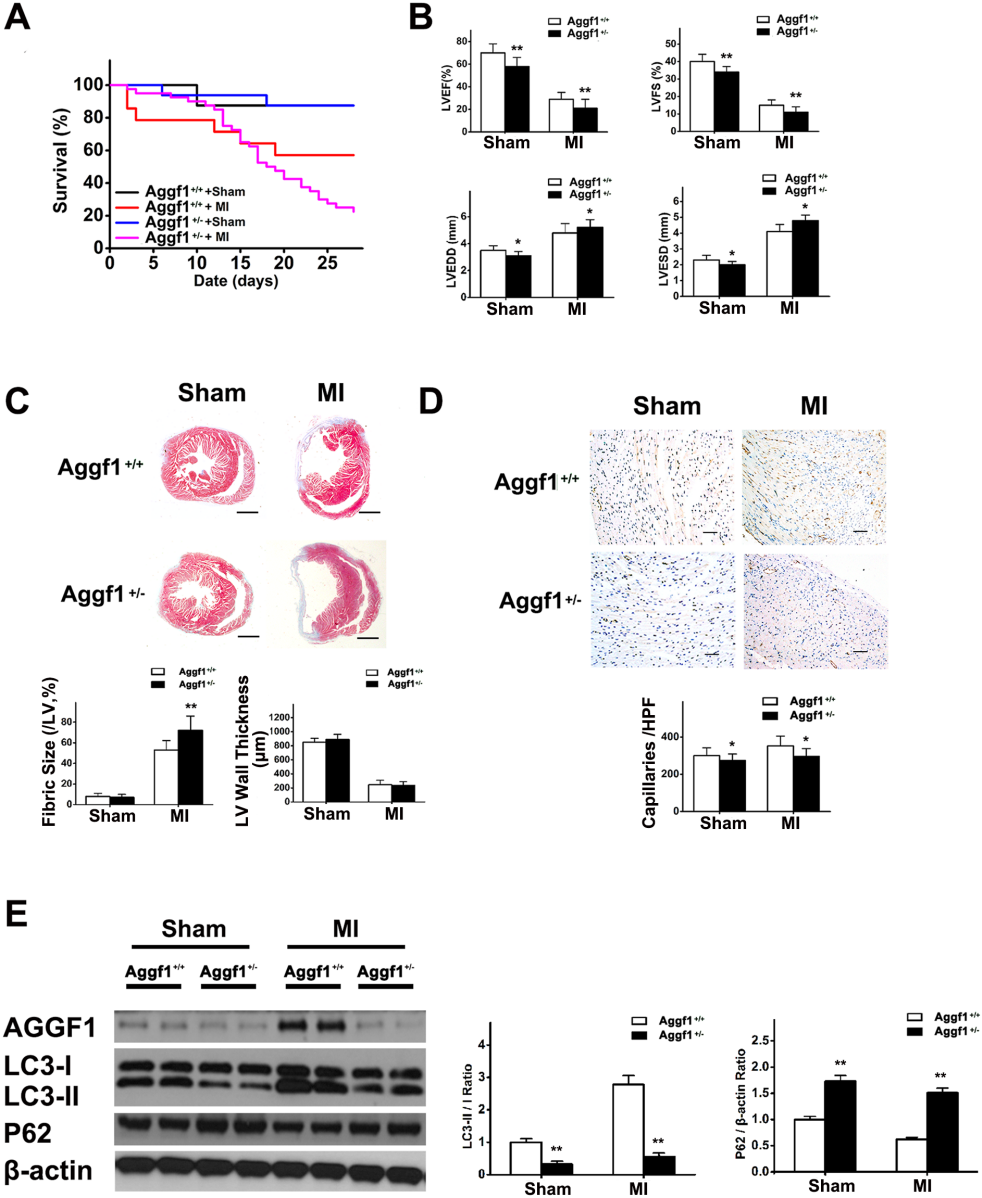
Reduced autophagy, decreased survival, larger infarct areas, compromised cardiac function and contraction, and impaired angiogenesis in *AGGF1*^*+/-*^ knockout mice after MI. **(A)** Four-week survival of *AGGF1*^*+/-*^ mice (*n* = 40) and *AGGF1*^*+/+*^ mice (*n* = 14) under MI as compared to *AGGF1*^*+/-*^ mice (*n* = 16) and *AGGF1*^*+/+*^ mice (*n* = 8) with sham operation. **(B)** Cardiac function, contraction, and structure were impaired in *AGGF1*^*+/-*^ mice compared to wild-type mice with or without LAD ligation. **(C)** Masson trichrome staining of cross-sections of hearts to measure fibrotic sizes of LV and LV wall thickness (*n* = 5/group). Scale bar = 1 mm. **(D)** CD31 immunostaining of myocardial sections 28 d after ligation (*n* = 5/group). Scale bar = 50 μm. **(E)** Western blot analysis for autophagy markers in *AGGF1*^*+/-*^ mice (*n* = 6) and *AGGF1*^*+/+*^ mice (*n* = 6) after MI or sham operation. Underlying data are shown in S1 Data.

To confirm whether AGGF1 could promote autophagy, leading to increased survival after MI, we created an MI model using *Aggf1*^*+/-*^ mice. The survival rate of *Aggf1*^*+/-*^ mice decreased 4 wk after MI compared with wild-type mice (Fig 6A). Echocardiography showed that LVEF and LVFS were significantly lower in *Aggf1*^*+/-*^ mice than in wild-type mice with or without MI (Fig 6B), indicating a compromised myocardial contraction by AGGF1 haploinsufficiency. Compared with wild-type mice, LVEDD and LVESD decreased in *Aggf1*^*+/-*^ mice with sham operation but increased in mice with LAD ligation (Fig 6B). The infarct area (fibrotic size) after MI was larger in *Aggf1*^*+/-*^ mice than in wild-type mice (Fig 6C). The CD31-positive vessel density was significantly less in *Aggf1*^*+/-*^ mice than in wild-type mice with or without MI (Fig 6D), indicating a critical role of AGGF1 in angiogenesis in vivo.

To identify the mechanism for the reduced angiogenesis, reduced survival, and compromised cardiac function and contraction in *Aggf1*^*+/-*^ mice, we analyzed autophagy in these mice. After MI, the expression level of LC3-II was reduced, whereas p62 expression was increased in *Aggf1*^*+/-*^ mice compared to wild-type mice (Fig 6E), indicating a correlation between reduced angiogenesis and inhibition of autophagy in *Aggf1*^*+/-*^ mice.

### Autophagy Is Required for AGGF1-Mediated Angiogenesis and Cardiac Repair after MI: An Autophagy Inhibitor Study

**Fig 7:**
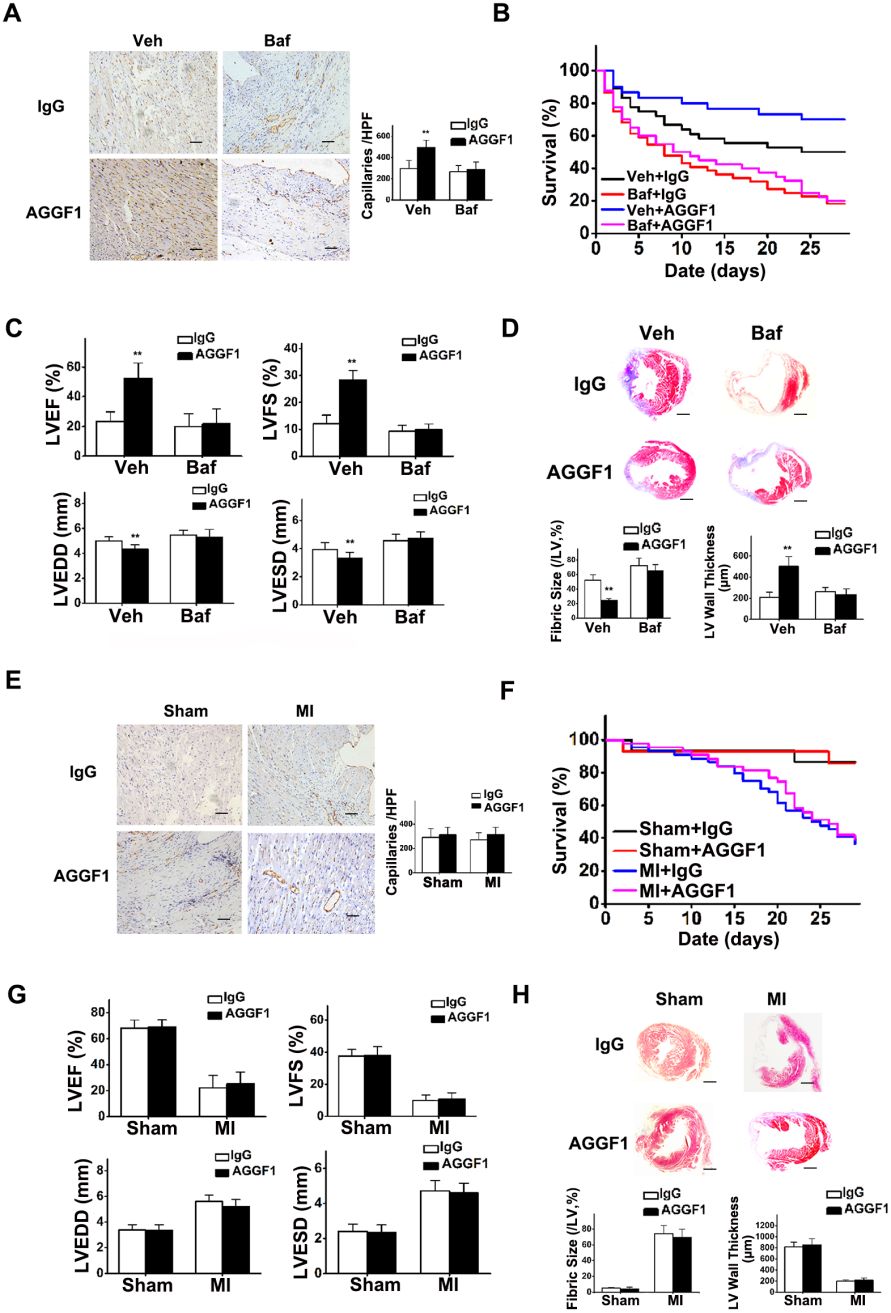
Autophagy is required for AGGF1-induced therapeutic angiogenesis, survival, and recovery of myocardial functions and contraction of MI mice. **(A)** Bafilomycin A1 (Baf) blocked AGGF1-induced angiogenesis (CD31 immunostaining) in heart sections 28 d after treatment (*n* = 5/group). Scale bar = 50 μm. **(B)** Four-week survival of MI mice under different treatments (MI + Veh + IgG, n = 36; MI + Veh + AGGF1, n = 30; MI + Baf + IgG, n = 44; MI + Baf + AGGF1, n = 40). **(C)** Effects of AGGF1 protein therapy with and without bafilomycin A1 pretreatment on the function of the left ventricle (MI + Veh + IgG, n = 18; MI + Veh + AGGF1, n = 21; MI + Baf + IgG, n = 8; MI + Baf + AGGF1, n = 8). **(D)** Effects of AGGF1 protein therapy with and without bafilomycin A1 pretreatment on the cardiac fibrosis (Masson trichrome staining of cross-sections) and LV wall thickness 4 wk after treatment (*n* = 5/group). Scale bar = 1 mm. **(E)** CD31 immunostaining in autophagy-deficient *Becn1*^*+/-*^ mice after LAD ligation (*n* = 5/group). Scale bar = 50 μm. **(F)** AGGF1 protein therapy showed no effect on 4-wk survival after MI of *Becn1*^*+/-*^ mice (Sham + IgG, n = 15; Sham + AGGF1, n = 14; MI + IgG, n = 44; MI + AGGF1, n = 43). **(G)** Echocardiographic parameters at 4 wk after MI. Effects of AGGF1 protein therapy on myocardial functions and contraction in *Becn1*^*+/-*^ mice (Sham + IgG, n = 13; Sham + AGGF1, n = 12; MI + IgG, n = 16; MI + AGGF1, n = 17). **(H)** Effects of AGGF1 protein therapy on the cardiac fibrosis 4 wk after MI in *Becn1*^*+/-*^ mice (*n* as in A). Scale bar = 1 mm. Underlying data are shown in S1 Data.

Because autophagy is required for AGGF1-promoted EC proliferation, migration, and capillary tube formation as demonstrated above, we hypothesized that autophagy is essential for AGGF1-mediated angiogenesis and recovery of myocardial function in mice after MI. To test this hypothesis, we carried out a study with bafilomycin A1 in mice. Immunostaining with CD31 showed that AGGF1 protein therapy increased CD31-positive vessel density compared with IgG treatment (compare AGGF1 and IgG in the vehicle group, *p* < 0.01), but bafilomycin A1 treatment eliminated the angiogenic effect of AGGF1 treatment (compare AGGF1 and IgG in the Baf group, *p* > 0.05) (Fig 7A). These data indicate that autophagy is required for angiogenesis in vivo.

AGGF1 protein therapy increased the survival rate of MI mice compared to IgG treatment in the vehicle group (Fig 7B). However, preconditioning by daily intraperitoneal injection of bafilomycin A1 (0.3 mg/kg) for five consecutive days after MI and before AGGF1 treatment eliminated the pro-survival function of AGGF1 (compare AGGF1 and IgG in the Baf group, Fig 7A).

Bafilomycin A1 eliminated the therapeutic effects of AGGF1 on recovery of LVEF, LVFS, LVEDD, and LVESD (Fig 7C and S14 Fig). H&E staining with Masson trichrome showed that AGGF1 treatment reduced the infarct size and increased LV wall thickness in the infarct areas compared to IgG treatment (the vehicle group, Fig 7D), but bafilomycin A1 treatment blocked the therapeutic effects of AGGF1 on cardiac fibrosis (the Baf group, Fig 7D).

### Autophagy Is Required for AGGF1-Mediated Angiogenesis and Cardiac Repair after MI: A Study with Autophagy-Deficient Becn1^+/-^ Mice

To further test the hypothesis that autophagy is essential for AGGF1-mediated angiogenesis and recovery of myocardial function after MI, we assessed the effects of AGGF1 protein in an autophagy-compromised background in mice. Beclin 1, a mammalian homology of yeast Atg6/Vps30, is an initiator of autophagy and is required for autophagosome formation [16]. Heterozygous *Becn1*^*+/-*^ mice showed defective autophagy [17]. Immunostaining with CD31 showed that AGGF1 did not have any effect on angiogenesis (CD31-positive vessel density) in *Becn1*^*+/-*^ mice either with sham operation or after MI surgery (Fig 7E). These data indicate that without autophagy, the angiogenic activity of AGGF1 is completely lost, suggesting that autophagy is essential for angiogenesis.

As shown in Fig 7F, AGGF1 protein therapy did not affect the survival rate of MI in *Becn1*^*+/-*^ mice compared to IgG treatment. MI mice with IgG treatment (*n* = 44) demonstrated a 79.54% 2-wk survival rate, whereas MI mice with AGGF1 treatment (*n* = 43) had an 83.72% 2-wk survival rate (*p* = 0.34) (Fig 7F). At 4 wk, there remained no significant difference between the survival rates of *Becn1*^*+/-*^ MI mice with AGGF1 treatment (39.53%) and with IgG treatment (36.36%) (*p* = 0.28). These data suggest that the effectiveness of AGGF1 protein therapy on MI survival requires beclin 1 and autophagy.

Echocardiography showed that compared to IgG treatment, AGGF1 treatment did not improve LVEF, LVFS, LVEDD, LVESD, cardiac fibrosis, infarct sizes, and LV wall thickness in *Becn1*^*+/-*^ mice (Fig 7G and 7H). Altogether, these data indicate that beclin 1 and autophagy are essential for therapeutic recovery of myocardial functions after MI with AGGF1 protein therapy.

### Autophagy Is Required for AGGF1-Mediated Angiogenesis and Cardiac Repair after MI: A Study with Autophagy-Deficient *Atg5*^*flox/flox*^ Mice

To further validate the critical role of autophagy in AGGF1-mediated angiogenesis and cardiac repair, we performed AGGF1 therapy for MI in mice lacking *Atg5* expression in the myocardium. *Atg5* forms a complex with Atg12 and Atg16L1, which can act as an E3 ligase to facilitate conjugation of LC3 to phosphaidylethanolamine in the autophagic membrane. We injected adenoviruses with AAV9-CMV-Cre into the myocardium of *Atg5*^*flox/flox*^ mice to generate *Atg5* KO mice (AAV9-GFP as control). Western blot analysis with cardiac extracts showed that *Atg5* expression is absent and the level of autophagy was low in *Atg5* KO mice 2 wk after virus injection compared with AAV-GFP mice (S15 Fig).

**Fig 8:**
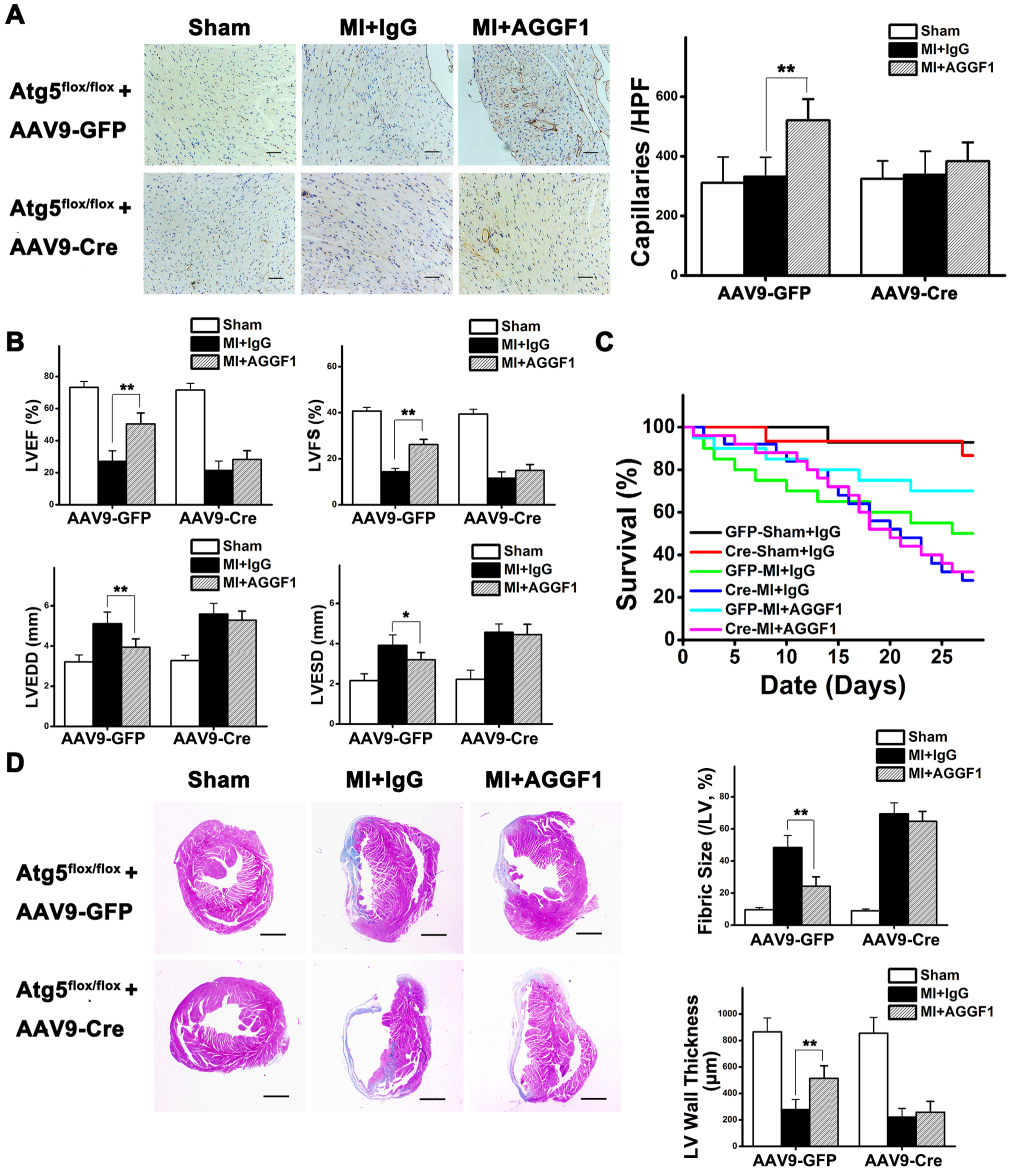
*Atg5* KO blunted AGGF1-induced therapeutic angiogenesis, survival, and recovery of myocardial functions and contraction of MI mice. **(A)** AGGF1-induced angiogenesis (CD31 immunostaining) in heart sections 28 d after MI in AAV9-GFP mice. *Atg5* KO blocks AGGF1-induced angiogenesis (CD31 immunostaining signal) in heart sections 28 d after treatment (*n* = 5/group, AAV9-CMV-Cre *Atg5* KO mice). Scale bar = 50 μm. (**B**) Four-week survival of MI mice under different treatments (Sham + AAV9-GFP + IgG, n = 14; MI + AAV9-GFP + IgG, n = 20; MI + AAV9-GFP + AGGF1, n = 20; Sham + AAV9-Cre + IgG, n = 15; MI + AAV9-Cre + IgG, n = 25; MI + AAV9-Cre + AGGF1, n = 25). (**C**) Effects of AGGF1 protein therapy under different treatments on the function of the left ventricle (Sham + AAV9-GFP + IgG, n = 13; MI + AAV9-GFP + IgG, n = 10; MI + AAV9-GFP + AGGF1, n = 14; Sham + AAV9-Cre + IgG, n = 13; MI + AAV9-Cre + IgG, n = 7; MI + AAV9-Cre + AGGF1, n = 8). (**D**) Effects of AGGF1 protein therapy under different treatments on cardiac fibrosis (Masson trichrome staining of cross-sections) and LV wall thickness 4 wk after treatment (*n* = 5/group). Scale bar = 1 mm. Underlying data are shown in S1 Data.

MI was created in *Atg5* KO mice and control mice. In AAV9-GFP control mice, AGGF1 protein therapy significantly increased the density of CD31-positive vessels in the heart after MI compared with IgG treatment (*p* < 0.01) (Fig 8A). In AAV9-CMV-Cre *Atg5* KO mice with deficient autophagy, AGGF1 protein therapy failed to increase the density of CD31-positive vessels in the heart after MI compared with IgG treatment (*p* > 0.05) (Fig 8A). These results further demonstrate that autophagy is required for angiogenesis in vivo.

In AAV9-GFP control mice, AGGF1 treatment increased the survival rate of MI mice compared to IgG treatment (Fig 8B). However, the therapeutic effect of AGGF1 was completely lost in AAV9-CMV-Cre *Atg5* KO mice with deficient autophagy (Fig 8B).

Echocardiography showed that AGGF1 treatment significantly increased LVEF and LVFS, reduced LVEDD, LVESD, cardiac fibrosis, and infarct sizes, and increased LV wall thickness in AAV9-GFP control mice with MI compared with IgG treatment (Fig 8C and 8D), but the therapeutic effect of AGGF1 was completely lost in AAV9-CMV-Cre *Atg5* KO mice with deficient autophagy (Fig 8C and 8D). Together, these data indicate that autophagy is essential for therapeutic recovery of myocardial functions after MI with AGGF1 protein therapy.

### Molecular Signaling Pathway for AGGF1-Induced Autophagy

**Fig 9:**
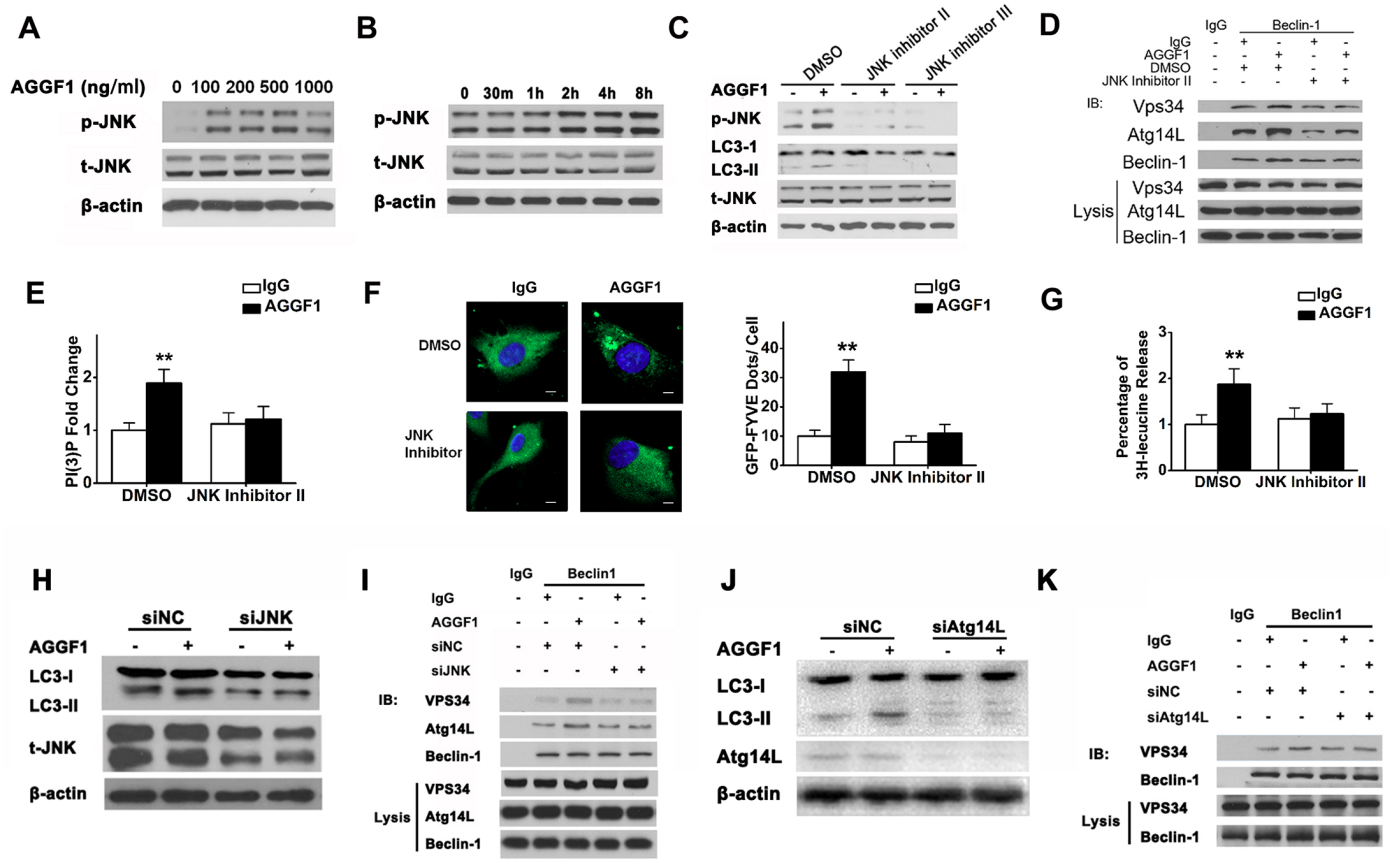
Molecular signaling pathway for AGGF-induced autophagy. **(A)** Western blot analysis for phosphorylation of JNK with protein extracts from HUVECs treated with different concentrations of AGGF1 for 4 h. **(B)** Activation of JNK in HUVECs treated with AGGF1 protein (500 ng/ ml) for different time points. **(C)** JNK inhibitors II and III inhibited AGGF1-induced autophagy (LC3-II levels) and activation of JNK. **(D)** Co-immunoprecipitation to assess the assembly of Beclin1-Vps34-Atg14L complex. Immunoprecipitation, beclin 1 antibody, or control IgG; Western blot, individual antibodies for Vsp34, Atg14L, or beclin 1. **(E)** Vps34 lipid kinase activity with ELISA assays (*n* = 3/group). **(F)** Autophagic activity assay for membrane-associated PI(3)K (GFP-2xFYVE dots). HUVECs were transfected with GFP-2xFYVE for 24 h and then treated with JNK inhibitor II or control DMSO, followed by treatment with AGGF1 or IgG (*n* = 6/group). Scale bar = 10 μm. **(G)** Autophagic activity assay for degradation of long-lived, damaged proteins by ^3^H-leucine release in HUVECs (*n* = 6/group). (**H**) *JNK1* siRNA inhibited AGGF1-induced autophagy (LC3-II levels) and expression of JNK1. (**I**) Co-immunoprecipitation to assess the assembly of the Beclin1-Vps34-Atg14L complex by *JNK1* siRNA. (**J**) *Atg14L* siRNA inhibited AGGF1-induced autophagy (LC3-II levels) and expression of Atg14L. (**K**) Co-immunoprecipitation to assess the assembly of the Beclin1-Vps34-Atg14L complex by *Atg14L* siRNA. Underlying data are shown in S1 Data.

To identify the molecular signaling pathway for autophagy induced by AGGF1, we analyzed the activation/phosphorylation of c-Jun N-terminal protein kinase (JNK). AGGF1 markedly induced JNK activation in HUVECs in a dose-dependent manner, with peak effect at a dose of 500 ng/ml (Fig 9A). Moreover, AGGF1 induced phosphorylation of JNK in a time-dependent manner (Fig 9B). Interestingly, as time increased, AGGF1-induced JNK phosphorylation increased (Fig 9B). Two different JNK inhibitors, JNK inhibitor II and JNK inhibitor III, blocked JNK activation by AGGF1. Consistent with this finding, AGGF1 increased the rate of autophagy as indicated by the increased level of LC3-II; however, the two JNK inhibitors inhibited autophagy induced by AGGF1 (Fig 9C).

The Becn1-Vps34-Atg14L complex is involved in autophagy [12]. Since beclin 1 is required for autophagy by AGGF1 as shown in *Becn1*^*+/-*^ mice, we analyzed the effect of AGGF1 on the assembly of the Becn-1-Vps34-Atg14L complex. Co-immunoprecipitation assays showed that AGGF1 increased the complex formation between beclin 1 and Vps34 and between beclin 1 and Atg14L (Fig 9D). JNK inhibitor II reduced the complex formation among beclin 1, Vps34, and Atg14L (Fig 9D).

Vps34 is a class III PI3K and the activity (leading to PtdIns3P production) is required for autophagosome formation [18]. Thus, we measured the activity of Vps34 lipid kinase. Compared with IgG, AGGF1 significantly increased the Vps34 lipid kinase activity as measured for conversion of PtdIns to PtdIns(3)P by an ELISA assay (Fig 9E). In the presence of JNK inhibitor II, the increased Vps34 PI3K activity by AGGF1 disappeared (Fig 9E). Moreover, in an assay for Vps34 PI3K-dependent intracellular localization of a GFP-FYVE reporter protein, predominantly localized to membranes of endocytic compartments (the FYVE domain preferably binds to Ptdlns(3)P to other phosphoinositides), AGGF1 treatment increased the number of GFP-2-FYVE dots, an indication of increased Vsp34 PI3K activity (Fig 9F). However, JNK inhibitor II significantly decreased the number of AGGF1-induced green GFP-2-FYVE dots (Fig 9F).

The last step in autophagy is the degradation of autophagic cargos in the lysosomes. Autophagic cargos are mostly long-lived proteins, and their rate of degradation, an indicator for autophagic flux, can be measured [18]. AGGF1 significantly increased the degradation of long-lived proteins in HUVECs, and this effect was blocked with JNK inhibitor II (Fig 9G).

To further confirm the involvement of the JNK pathway in AGGF1-mediated autophagy, we used siRNA to knock expression of *JNK1* down and determine the effect of AGGF1 on activation of autophagy. AGGF1 failed to increase the LC3-II/LC3-I ratio (activation of autophagy) when *JNK1* expression was knocked down (Fig 9H). *JNK1* knockdown also reduced the AGGF1-induced formation of the beclin1-Atg14L-VPS34 complex (Fig 9I). Combined with the data obtained on JNK inhibitors, these data demonstrate the involvement of the JNK pathway in AGGF-mediated autophagy.

Because Atg14L interacts with VPS34, we used siRNA to knock expression of *Atg14L* down and then determined the effect of AGGF1 on activation of autophagy. AGGF1 failed to increase the LC3-II/LC3-I ratio (activation of autophagy) when *Atg14L* expression was knocked down (Fig 9J). *Atg14L* knockdown also reduced the AGGF1-induced formation of the beclin1-Atg14L-VPS34 complex (Fig 9K). After autophagy activation, DFCP1, an Endoplasmic Reticulum (ER)-residing PI3P binding molecule, is recruited to a membrane compartment related to autophagosome biogenesis. We found that the number of DFCP1-positive puncta was increased by AGGF1 treatment in HUVECs (S16 Fig). Knockdown *Atg14L* significantly decreased the number of DFCP1 puncta and blocked the effect of AGGF1 (S16 Fig). These data suggest that AGGF1-induced PI-3K activation resulted in an increased level of PI3P, which was required for DFCP1 recruitment. As a whole, these data demonstrate that Atg14L is required for AGGF-mediated autophagy.

**Fig 10:**
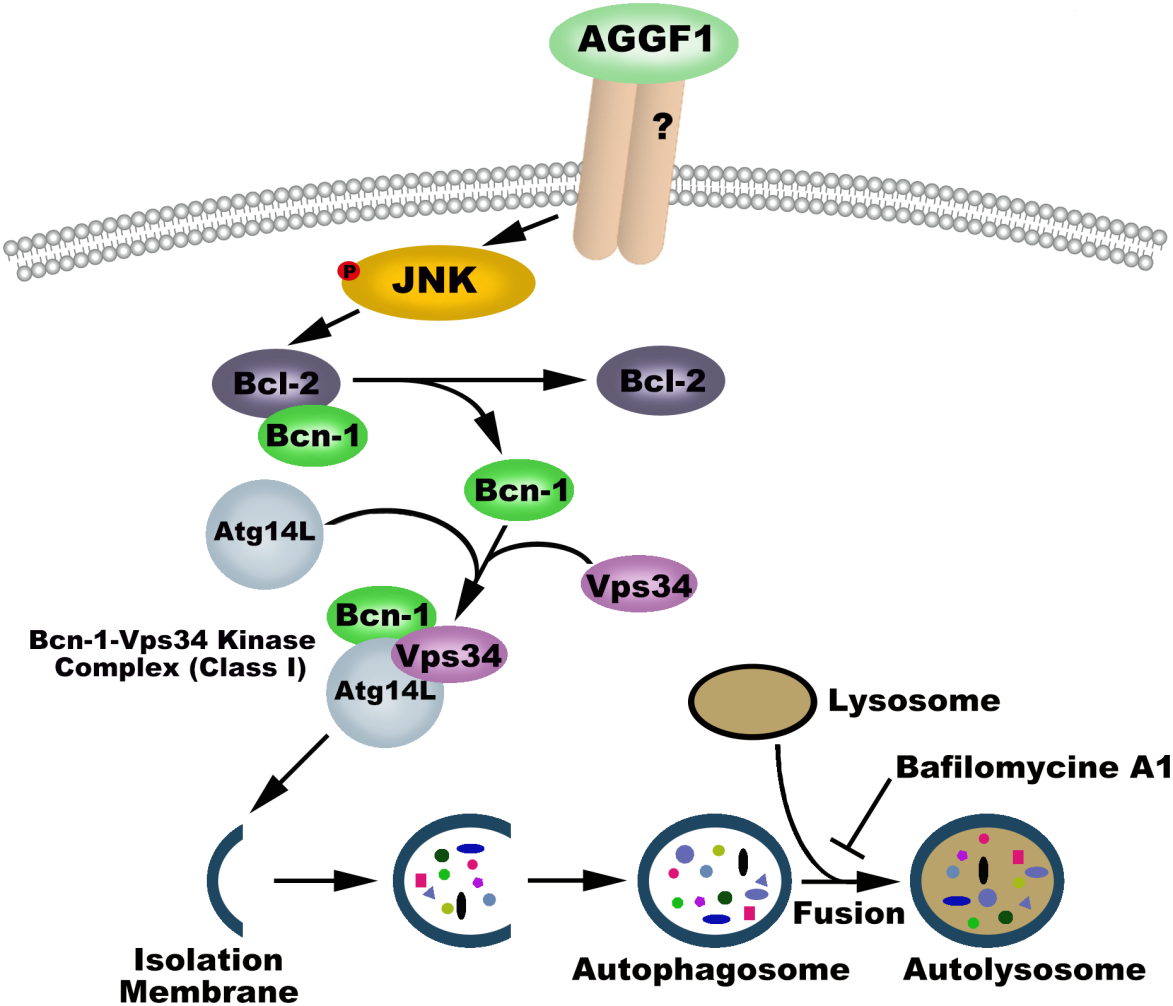
Schematic diagram showing the molecular signaling pathway for AGGF1-induced autophagy and angiogenesis.

The above data suggest that AGGF1 induces phosphorylation/activation of JNK, which increases the activity of Vps34 and the assembly of the Becn-1-Vps34-Atg14L complex, resulting in the formation of autophagosomes and onset and completion of autophagy (Fig 10).

## Discussion

In this study, we found that angiogenic factor AGGF1 can activate autophagy in ECs in vitro and in MI mice in vivo, thereby identifying a new and critical upstream regulator of autophagy. Interestingly, angiogenic factor AGGF1 has been found to induce autophagy during angiogenesis. First, we demonstrated that mice with acute MI (subject to LAD) showed an increased expression level of either *AGGF1* mRNA or protein, which was associated with up-regulated autophagy (Fig 4). Second, autophagy was inhibited in heterozygous *Aggf1*^*+/-*^ knockout mice (Fig 6). Third, treatment of HUVECs with recombinant AGGF1 dramatically induced autophagy (Fig 1). These data indicate that AGGF1 is a strong promoter for autophagy. Identification of the important role of an angiogenic factor in autophagy is a novel aspect of the study. The data also revealed a novel in vivo function for AGGF1 in the regulation of autophagy. Mechanistically, we found that AGGF1 activates autophagy by stimulating JNK activation. Two JNK inhibitors and *JNK1* siRNA blocked induction of autophagy by AGGF1 (Fig 9). AGGF1-induced JNK activation increased the activity of Vps34 and facilitated co-assembly of the Becn-1-Vps34-Atg14L complex required for autophagy. Consistently, these functions were disrupted by JNK inhibitors and *JNK1* and *Atg14L* siRNAs (Fig 9). We have previously reported that AGGF1 activates AKT to regulate specification of veins in zebrafish [6]. However, increased AKT activation was shown to inhibit autophagy [19]. Therefore, it is the AGGF1-JNK signaling pathway, not the AGGF1-AKT signaling pathway, that modulates autophagy. This conclusion is supported by a recent finding that activation of JNK1 by FGF18 induced autophagy in bone growth [20].

During the initiation of autophagy, several other key modulators of the Becn-1-Vps34-Atg14L complex were identified, including Unc-51 Like Autophagy Activating Kinase 1 (ULK1) and Autophagy And Beclin 1 Regulator 1 (AMBRA1) [21]. Future studies may examine whether ULK1 or AMBRA1 affects the regulation of the complex of BECN-VPS34-Atg14 by the AGGF1-JNK pathway. Atg14L was shown to be a unique subunit of the autophagy-specific PI3K complex involved in autophagosome formation. Atg14L cooperated with the ER-resident Soluble N-ethylmaleimide-sensitive factor Attachment protein Receptor (SNARE) protein syntaxin 17 (STX17) to assemble autophagosome at ER-mitochondria contact sites [22]. It may be interesting to investigate whether AGGF1 modulates the Atg14L-STX17 interaction involved in autophagosome formation.

Another major novelty of this work involves the unexpected finding that autophagy is essential for therapeutic angiogenesis. The data in the present study demonstrated the essential role of autophagy in angiogenesis in vivo in mice. The molecular mechanism by which autophagy activates angiogenesis is unknown. One possible mechanism is that autophagy produces metabolites which are required for efficient EC proliferation, migration, and angiogenesis. Recently, fatty acid carbons and deoxyribose nucleoside triphosphate (dNTP) synthesis were shown to be required for EC proliferation and vascular sprouting [23].

Interestingly, AGGF1 activates autophagy not only in ECs, but also in all cells examined, including cardiac cells HL1 and H9C2 cells and vascular smooth muscle cells (VSMCs). These data suggest that the AGGF1 has a much broader biological function. Because the function of AGGF1 is thought to be more related to angiogenesis, the present study focused exclusively on ECs. The dramatic therapeutic effects of AGGF1 on mice with CAD and MI may be related to the beneficial effects of AGGF1-activated autophagy not only in ECs, but also in cardiomyocytes and other cells. Future studies with EC-specific, cardiomyocyte-specific, and VSMC-specific knockout mice may distinguish specific roles of AGGF1 in ECs, cardiomyocytes, and other cells.

Autophagy occurs at a low basal level under physiological conditions in the heart and is up-regulated under ischemia/reperfusion [24,25]. The basal level of autophagy in the heart plays an important role in maintaining cellular homeostasis; however, the role of increased autophagy in response to ischemia/reperfusion is controversial [12]. To date, it is not clear whether autophagy is protective (cell survival) or detrimental (cell death) to the heart during ischemia/reperfusion [13,25]. In some cases, up-regulation of autophagy was associated with a cardio-protective effect, but the cause–effect relationship was not conclusively demonstrated. In other cases, up-regulation of autophagy in response to ischemia/reperfusion was detrimental and exacerbated cardiomyocyte death [12]. In this study, we provide evidence to demonstrate that autophagy is essential for functional recovery of the heart from acute MI in mice. Preconditioning of MI mice with autophagy inhibitors bafilomycin A1 and chloroquine eliminated the therapeutic effects of AGGF1 protein on increased survival and improved myocardial contraction and recovery on cardiac structure (Fig 7). Similarly, AGGF1 protein completely lost its therapeutic effects in autophagy-deficient mice (*Becn1*^*+/-*^ knockout mice and AAV9-CMV-Cre *Atg5* KO mice) (Figs 7 and 8). These data suggest that autophagy plays a protective role under ischemia and is required for cardiac repair after acute MI. During the MI survival studies of *Becn1*^*+/-*^ knockout mice and AAV9-CMV-Cre *Atg5* KO mice, we made another interesting observation. Autophagy deficiency in *Becn1* KO and *Atg5* KO mice increased the survival of MI mice at the first 2 wk, but dramatically reduced MI survival starting after the first 2 wk (Figs 7 and 8). The data suggest that autophagy is protective to MI survival in the long run, and reduced autophagy leads to worsened survival. However, in the short run, autophagy may be damaging to MI survival, potentially due to strong stress response to ischemia. Instead, reduced autophagy leads to improved survival.

The use of angiogenic factors for generating new blood vessels from existing vasculature, i.e., therapeutic angiogenesis, has been proposed as an attractive strategy for treatment of CAD and MI patients. The VEGF (vascular endothelial growth factors) family of growth factors and fibroblast growth factor 2 (FGF2) are the widely studied angiogenic factors for therapeutic myocardial angiogenesis. For VEGF, the therapeutic dose was difficult to establish as lower doses did not show therapeutic effect, whereas high doses caused aberrant vascular growth and vascular permeability. For FGF2, long-term effects were not observed. We have found that vascular permeability increased in *Aggf1*^*+/-*^ KO mice and AGGF1 administration blocked vascular permeability in mice. Therefore, AGGF1 protein therapy has a unique advantage for therapeutic angiogenesis over VEGFA. To date, no therapeutic angiogenesis treatment has been approved by the US FDA and many important issues must be resolved before therapeutic angiogenesis becomes a practical patient therapy. One solution is to identify other angiogenic factors to achieve robust therapeutic angiogenesis. In this study, we show that in a mouse model for acute MI, AGGF1 protein therapy significantly reduced mortality and dramatically improved overall cardiac function and myocardial contraction by inhibiting cardiac hypertrophy, reducing infarct size, and preventing cardiac apoptosis and fibrosis in vivo (Fig 4). AGGF1 protein therapy enhanced myocardial angiogenesis in the mouse model for acute MI (Fig 5B), an effect that is relevant to the dramatically improved myocardial function and contraction. Our data have demonstrated that angiogenic factor AGGF1 is a new growth factor with promising therapeutic potential in the treatment of CAD and acute MI.

One of the challenges for therapeutic angiogenesis is its efficacy. Considering the essential role of autophagy in therapeutic angiogenesis, maintaining autophagy is required for effective therapeutic angiogenesis, and increasing the levels of autophagy may be a potential strategy to robustly increase the efficacy of therapeutic angiogenesis. For example, utilization of pharmacological agents such as rapamycin [12] that increase autophagy may increase the efficacy of therapeutic angiogenesis for CAD and MI during AGGF1 protein therapy.

In conclusion, the present study demonstrates that autophagy is essential for therapeutic angiogenesis, suggesting that manipulation of autophagy may serve as a potential strategy to robustly boost the efficacy of therapeutic angiogenic therapy for MI and other ischemic diseases. We show that AGGF1 activates autophagy, whereas haploinsufficiency of *AGGF1* inhibits autophagy. AGGF1 increases survival of mice with acute MI, reduces infarct areas, inhibits cardiac apoptosis and fibrosis, and leads to dramatic recovery of left ventricular function and myocardial contraction. Inhibition of autophagy by an autophagy inhibitor bafilomycin A1 or in *Becn1*^*+/-*^ and *Atg5* KO mice with deficient autophagy eliminated all therapeutic effects of AGGF1. Together, these data uncover new fundamental molecular mechanisms underlying autophagy and therapeutic angiogenesis and provide a novel treatment strategy for CAD and MI, the leading causes of sudden death worldwide.

## Materials and Methods

### Ethics Statement

Both animal care and experimental procedures were performed according to the guidelines by the National Institutes of Health (NIH) and the Guide for the Care and Use of Laboratory Animals by the National Research Council of the United States of America and approved by the Institutional Animal Care and Use Committee (IACUC) of Cleveland Clinic (2012–0899) and according to the Guide for the Care and Use of Animals for Research by the Ministry of Science and Technology of the P. R. China (2006–398) and approved by the Ethics Committee on Animal Research of College of Life Science and Technology of Huazhong University of Science and Technology ([2014]IEC [S089]).

### Cell Culture, Transfection, Autophagy Inhibitors, Antibodies, and Western Blot Analysis

Human umbilical vein endothelial cells (HUVECs) were cultured in the EGM-2 medium containing 5% (v/v) fetal bovine serum (FBS) and EGM-2 Single Quots (Lonza). A polyclonal antibody against AGGF1 was from Proteintech. Antibodies against CD31, LC3, p62, cleaved caspase-3, Bcl-2, Bax, phospho-JNK, total JNK, Vps34, Atg14L, beclin-1, and β-actin were from Cell Signaling. The antibody against cleaved poly (ADP-ribose) polymerase (PARP) was from Santa Cruz.

Cardiac endothelial cells (ECs) were isolated from *AGGF1*^*+/-*^ or wild-type mice as described previously [26] and cultured as for HUVECs described above.

HUVECs were transfected with siRNA using the electroporation P5 Primary Cell Nucleofector Kits (Lonza). The transfection efficiency of siRNA was determined by real-time RT-PCR analysis of a target gene. Cells were discarded if contaminated by mycoplasma or other microorganisms.

HUVECs were treated with 25 μM chloroquine (Sigma) or 100 nM bafilomycin A1 (Sigma) for 1 h before AGGF1 or IgG treatment. The dosage for autophagy inhibitors was as reported previously [27,28].

Western blot analysis was carried out with different antibodies as described previously [29,30]. In brief, 4 wks after treatment, mice were anesthetized and left ventricles were dissected out for extraction of total proteins. Cultured HUVECs were washed three times with PBS and used for extraction of total proteins. Proteins were extracted on ice in 20 mM Tris-HCl, pH 7.6, 150 mM NaCl, 0.1% DOC, 0.5% NP-40, 10% glycerol, 1 mM glycerophosphate, 1 mM NaF, 2.5 mM Na pyrophosphate, 1 mM Na3VO4, and a cocktail of protease inhibitors (Calbiochem). Extracted proteins were then mixed with reducing laemmLi sample buffer, boiled for 10 min, separated by SDS-polyacrylamide gel electrophoresis (PAGE), transferred to nitrocellulose membranes, and blotted with a primary antibody and appropriate secondary antibodies. Images from western blot were captured and quantified using 1-D Analysis Software and Quantity One (Bio-Rad).

### Protein Purification

The full-length human *AGGF1* cDNA was cloned into a bacterial expression vector pET-28b (Novagen), resulting in an overexpression construct for 6xHis-tagged AGGF1, pET-28-AGGF1 [2]. The pET-28-AGGF1 expression construct was transformed into *Escherichia coli* BL21 (DE3) Star, and 6xHis–AGGF1 protein was overexpressed and purified using a Ni-NTA agarose column according to the manufacturer's instructions (Qiagen). The eluted protein was dialyzed, and quality of purification was examined by SDS-PAGE by coomassie blue staining and western blot analysis using an anti-AGGF1 antibody [2].

### Mouse Models for Myocardial Infarction and AGGF1 Protein Therapy

Male C57BL/6 mice were used for all studies. The *Aggf1*^*+/-*^ KO mice with a gene-trap allele were created by us. The autophagy-deficient *Becn1*^*+/-*^ KO mice (*Becn1*^*tmeBlev*^) were from the Jackson Laboratory. *Atg5*^*flox/flox*^ mice [31] were kindly provided by Dr. Noboru Mizushima at University of Tokyo, RIKEN BRC, and Dr. Quan Chen at the Chinese Academy of Sciences. To create *Atg5* KO in mice, *Atg5*^*flox/flox*^ mice were anesthetized and injected with 0.2 ml of the adenovirus AAV9-GFP or AAV9-CMV-Cre (Vector Biolabs) into the left ventricular myocardium using a 35-gauge needle at multiple sites. Animal care and experimental procedures were approved by the Institutional Animal Care and Use Committee (IACUC) of Cleveland Clinic and the Ethics Committee on Animal Research of College of Life Science and Technology at Huazhong University of Science and Technology.

The acute MI model was created by ligation of the left anterior descending (LAD) coronary artery as described previously [32] with male mice at the age of 10–12 wk (about 25 g). The mice were anesthetized with an intraperitoneal injection of sodium pentobarbital (50 mg/kg) and then intubated using a fine polyethylene cannula connected to a small animal ventilator. The body temperature of the mouse was monitored using a rectal sensor at all times during the surgical procedure and maintained by a heated surgical plate. After respiration of the mouse was controlled by the ventilator, a thoracotomy incision was made in the second intercostal space, and the heart was exteriorized out of the chest. The LAD coronary artery was ligated permanently with a 7–0 nonabsorbable surgical suture and the heart was then returned inside the chest. The chest wall was closed in layers and skin incision was closed by sutures. The mouse was then removed from the ventilator and kept warm in a cage at 37°C overnight. Sham-operated mice were subjected to the same surgical treatment, but the LAD was not ligated.

C57BL/6N mice were assessed for baseline cardiac function with echocardiography prior to surgery and then subjected to LAD ligation. One day after the surgery, each animal was examined with echocardiography to confirm the success of the MI surgery. One week after the surgery, the mice were injected intravenously with AGGF1 (0.25 mg/kg body weight) or the same dose of nonimmune control IgG (0.25 mg/kg body weight, R&D systems) twice a week for 2 wk or until death. The IgG was used as the control as reported in other studies [33–35] and was considered to be a better control than saline without any protein.

In the set of experiments for bafilomycin A1 (an autophagy inhibitor), we divided the mice that survived the LAD ligation procedure for 1 wk and were ensured to have successful MI by echocardiography into four groups: an IgG-treated group with vehicle pretreatment (IgG with 0.1% DMSO), an IgG-treated group with pretreatment with bafilomycin A1 (0.3 mg/kg body weight, Sigma), an AGGF1-treated group with vehicle pretreatment, and an AGGF1-treated group with pretreatment with bafilomycin A1. Bafilomycin A1 is a vascular H^+^-ATPase inhibitor and a membrane-permeable lysosomal inhibitor that blocks autophagosome-lysosome fusion to prevent the final digestion step in the process of autophagy [36]. In these experiments, one day after surgery, the mice were assessed by echocardiography for the success of the MI procedure and then treated with bafilomycin A1 or vehicle control by daily intraperitoneal injection for 5 d. One week after the surgery, the AGGF1 protein or control IgG was then injected through tail vein twice a week for 2 wk. All mice were randomized in all experimental protocols.

### Echocardiography

Echocardiography was performed with a Vevo 2100 High-Resolution Micro-Ultrasound System (Visual Sonics Inc., Toronto, Canada) at five different time points: pre-surgery, 1 d after LAD ligation and administration of AGGF1 protein or IgG (1 d), 1 wk, 2 wk, and 4 wk after the surgery to determine the baseline heart function and ventricular dimensions in different experimental groups of mice.

The mouse under study was anesthetized with 1% isoflurane, placed on a heat pad in the supine position, and kept at 37°C to minimize confounding of data by fluctuating body temperatures. Hairs were removed by depilatory cream on the left chest before echocardiography. A 30 MHz variable frequency transducer was used to capture two-dimensional echocardiographic images of the mid-ventricular short axis and parasternal long axes when the heart rate of the mouse was between 450 and 550 beats per minute. Echocardiographic analysis was performed with digital images using a standard formula as previously described [14,15]. All procedures of echocardiography, including data acquisition and analysis, were performed by a researcher who was blind of the experimental treatments to avoid biases.

### Immunohistological Analysis

Four weeks after AGGF1 or IgG treatment, mice were anesthetized, euthanized, and the hearts were excised and fixed overnight. The fixed hearts were sectioned and immunohistochemical staining was performed on paraffin-embedded sections with a primary antibody against AGGF1 or CD31, which was followed by incubation with a biotinylated secondary antibody as described previously [29,37]. The sections were then treated with peroxidase-conjugated biotin–avidin complex using VECTASTAIN ABC-AP and visualized by DAB. Slides were counterstained with H&E staining. Vessel density was evaluated by counting the number of neovessels and arterioles in five random and non-repeated high power fields.

### Myocardial Apoptosis

Myocardial apoptosis was measured first by the TUNEL assay (terminal deoxynucleotidyltransferase (Tdt)-mediated dUTP nick-end labeling) [29]. Four weeks after treatments with AGGF1 or IgG control, mouse hearts were excised and fixed in 4% paraformaldehyde, embedded in paraffin, cut into 5 μm-thickness sections and used for the TUNEL assay using the In Situ Cell Death Detection kit (Roche Diagnostics GmbH). The images were visualized under a fluorescence microscope and captured. The border zone of fibrosis was examined carefully. More than five fields in three different sections were examined for each mouse by a researcher who was blinded to the treatments. The percentage of the number of TUNEL-positive cells over the total number of nuclei as determined by DAPI was calculated. Heart sections incubated with the label solution but without terminal transferase were used as negative controls. A TUNEL-positive control was included by incubating sections with DNase I (3000 U/ml in 50 mm Tris-HCl, pH 7.5, 1 mg/ml BSA) for 15 min at 15–25°C to induce DNA strand breaks prior to the labeling procedure.

Myocardial apoptosis was also measured by the caspase-3 activity [29]. We used a caspase-3 colorimetric assay kit and followed the manufacturer’s instruction (Promega). The absorbance of p-nitroaniline cleaved by caspase-3 was measured at 405 nm using FlexStation3. Data on the caspase-3 activity were standardized over the sham group and the fold change of the caspase-3 activity (relative caspase activity) was compared among different groups.

### Apoptosis and Proliferation of HUVECs

HUVECs were seeded on coverslips in 24-well plates. The AGGF1 protein at different concentrations or an equal dose of negative control IgG in PBS was added to the wells at the next day. After 2 h of incubation, HUVECs were incubated under a hypoxic condition with 1% O_2_. After 12 h, the coverslips were washed in PBS and fixed in 4% paraformaldehyde for 1 h at room temperature. After blocking and permeabilization, the slides were used for the TUNEL assay as described above.

HUVECs were seeded in 6-cm plates at 1 × 10^6^/ml. The AGGF1 protein at different concentrations or an equal dose of IgG in PBS was added to the wells at the next day. After hypoxic treatment, cells were lysed and the caspase-3 activity was determined using the caspase-3 fluorescence kit as described above.

Apoptosis of HUVECs was also assessed by western blot analysis using anti-cleaved caspase-3, anti-Bax, anti-Bcl-2, and anti-cleaved PARP as described above.

For cell proliferation, HUVECs were cultured in 96-well plates, incubated with AGGF1 or control IgG for 2 h, and cultured in a hypoxia chamber equilibrated with 1% O_2_ for different time points. The cells were then used for cell proliferation assays with the 2-(2-methoxy-4-nitrophenyl)-3-(4-nitrophenyl)-5-(2,4-disulfophenyl)-2H-tetrazolium, monosodium salt (WST-8) kit (Cell Counting Kit-8, Dojindo Laboratories). The number of living cells in triplicate wells was directly proportional to the amount of the WST-8formazan dye generated by measuring the absorbance at 450 nm.

### Cell Migration Assays

Two different types of cell migration assays were carried out as described by us [2]. The first is the scratch wound assay. HUVECs were grown in 6-well plates as a confluent monolayer and mechanically scratched with a pipette tip. The cells were incubated with EBM containing the AGGF1 protein (500 ng/ml) or control IgG and other treatment agents. Migration was quantified as the ratio of the area covered with cells over the cell-free area.

The second is the Transwell migration assay. HUVECs were plated into the upper compartment and were allowed to migrate towards AGGF1 (500 ng/ml) or IgG in the lower chamber (Corning Costar, MA, USA). After 6 h of incubation, HUVECs on the bottom of the Transwell membrane were fixed with 4% paraformaldehyde at 37°C for 20 min and stained with hematoxylin at 37°C for 5 min. The membranes were washed three times with PBS and photographed. The migrated cells on the bottom of the surface were counted in eight standardized fields.

### HUVEC Tube Formation

Approximately 5 × 10^4^ HUVECs in EBM were seeded onto matrigel (Corning) with the AGGF1 protein (500 ng/ml) or negative control IgG. After 6 h of incubation, 2 μg/ml of Calcein AM (Invitrogen, USA) was added directly to the well and incubated for 20 min. The well was washed with PBS three times. The images were visualized under a microscope and captured. The number of mature vessel tubes formed was counted as described previously [1,2]. Only completely closed tubes were regarded as mature tubes.

### *Ex Vivo* Mouse Aortic Ring Assays for Angiogenesis

The mouse aorta was removed under aseptic conditions. The dissected aorta was transferred to a dish containing cold EBM. To avoid contamination of other cell types, excessive fat tissue was quickly removed by forceps, and the aorta was then embedded into matrigel. The AGGF1 protein or PBS was added into the media. After 4 d of incubation in 37°C with 5% CO_2_, images of newly formed vessels were captured under a microscope and analyzed.

### Autophagy Assays for Vps34 Class III PI3 Kinase Activity

HUVECs were transfected with an expression plasmid for HA-tagged Vps34 using nucleofection (Lonza) and then incubated with different amounts of AGGF1 or control IgG. Exogenous Vps34 expressed in HUVECs was immunoprecipitated using an anti-HA antibody, measured using the class III PI3K assay, and was carried out using the Class III PI3-kinase ELISA kit (Echelon, UT, US).

HUVECs were transfected with an overexpression plasmid for pGFP-2xFYVE by nucleofection (Lonza). Twenty-four hours later, HUVECs were treated with or without JNK inhibitors for 1 h and then incubated with AGGF1 for 12 h. Images were captured with a florescence microscope and analyzed for the number of green GFP-FYVE dots with endocytic membrane Vps34 per cell.

### Assay for Autophagic Activity by Measurement of Degradation of Long-Lived Proteins

The assay was performed following the protocol described previously [18,38]. Briefly, HUVECs were plated and incubated with ^3^H-labeled leucine for 36 h. Then, cells were washed three times with PBS and incubated in unlabeled medium for 24 h to release short-lived proteins. After the chase period, cells were washed again three times with PBS and cultured in medium with AGGF1 or IgG for 12 h. The supernatant was collected and precipitated with trichloroacetic acid (TCA). The TCA-soluble fraction was measured by liquid scintillation counting. Total cell radioactivity was analyzed after lysis with 0.1 M NaOH. The degradation of long-lived protein was calculated as a percentage of the radioactivity in TCA-soluble fraction to the total cell radioactivity.

### Other Autophagy Assays

HUVECs were transfected with an overexpression plasmid for GFP-LC3B by nucleofection (Lonza). After 24 h of culture, HUVECs were treated with AGGF1 or control IgG for 12 h. Images were captured with a florescence microscope and analyzed for the ratio of green punctuate cells over all green cells with successful transfection. Autophagy was also monitored by electron microscopy as described previously [39].

### Real-Time RT-PCR Analysis

Total RNA was extracted from cultured cells or mouse hearts using Trizol (Invitrogen) according to the manufacturer’s instruction. A total of 0.5 μg of RNA samples was reverse-transcribed using M-MLV Reverse Transcriptase according to the manufacturer’s protocol (Promega). Quantitative real-time PCR analysis was then performed using the FastStart Universal SYBR Green Master (Roch) and a 7900 HT Fast Real-Time PCR System (ABI) as described previously [40–42]. Experiments were performed in triplicate.

### Data Analysis

Two-group comparisons were analyzed by a Student’s *t* test or nonparametric Wilcoxon rank test whenever appropriate (e.g., when the sample size was small and/or the distribution was not normal). For comparisons of more than two groups, one-way ANOVA or the generalized linear regression approach was employed for normal distributions and the Kruskal Wallis test for non-normal or small samples. Bonferroni correction was used to adjust for multiple testing after the overall F or Kruskal Wallis test showed statistical significance. The extract testing was used when the sample size was small (e.g., when all group sizes were <10). The survival curve analysis was analyzed using the Kaplan–Meier product-limit approach and compared by the log-rank test. Statistical significance is indicated by * *p* < 0.05 and ** *p* < 0.01.

## Supporting Information

S1 Data Excel file containing numerical values for all data. (XLS)

S1 Fig AGGF induces autophagy in HUVECs. Western blot analysis showed that administration of AGGF1 at different concentrations induced autophagy (increased LC3-II/β-actin and LC3-II/I ratios; decreased p62 expression). Serum starvation was used as positive control for autophagy activation.
(TIF)

S2 Fig AGGF1 activates autophagy in H9C2, HL1, and vascular smooth muscle cells (VSMCs). (TIF)

S3 Fig Increased cardiac apoptosis in *Aggf1*^*+/-*^ mice. Western blot analysis with apoptosis markers showed that the apoptosis pathway was increased in *Aggf1*^*+/-*^ hearts compared to wild-type samples.
(TIF)

S4 Fig CQ and Baf are effective in inhibition of autophagy. Western blot analysis showed that autophagy inhibitors CQ and Baf inhibited autophagy. Note that Baf inhibits the fusion of autophagosomes with lysosomes and thus inhibits autophagy although LC3-II expression is increased.
(TIF)

S5 Fig *Atg5* siRNA reduces the expression level of *Atg5* mRNA. Real-time RT-PCR analysis showed that *Atg5* expression was successfully knocked down in HUVECs by siRNA. Underlying data are shown in S1 Data.
(TIF)

S6 Fig Hypoxia induces up-regulation of AGGF1 expression in HUVECs. (A) Western blot analysis to measure the AGGF1 protein expression levels in HUVECs under hypoxia with 1% O_2_ for different time points. The data were from experiments independently repeated at least four times. (B) Quantitative real-time RT-PCR analysis to measure the expression levels of *AGGF1* mRNA in HUVECs under hypoxia with 1% O_2_ for different time points. The data were from experiments independently repeated at least four times. Underlying data are shown in S1 Data.
(TIF)

S7 Fig Hypoxia induces up-regulation of AGGF1 expression in cardiac ECs. Western blot analysis was used to measure the AGGF1 protein expression levels in ECs isolated from mouse hearts under hypoxia with 1% O_2_ at different time points. The images were quantified and plotted below. The data were from experiments independently repeated at least three times. Underlying data are shown in S1 Data.
(TIF)

S8 Fig Effects of AGGF1 protein therapy on cardiac functions and contraction at different time points in mice with acute MI. C57BL/6N mice were assessed for baseline cardiac function with echocardiography prior to surgery and then subjected to LAD ligation. One day after the surgery, each animal was examined with echocardiography to confirm the success of the MI surgery. One week after the surgery, the MI mice received AGGF1 or control IgG twice a week for 2 wk or until death. Mice with sham operation were used as controls. The mice were then studied by Vevo-2100 echocardiography. Underlying data are shown in S1 Data.
(TIF)

S9 Fig Effects of AGGF1 protein therapy on post-MI longitudinal strain and strain ratios. (A) Schematic diagram showing the myocardial regions marked from the paraternal long axis view. AA, apical anterior; MA, mid anterior; BA, basal anterior; AI, apical inferior; MI, mid inferior; BI, basal inferior. (B) AGGF1 protein therapy increased longitudinal strain in the global myocardial region or the infarcted region. Peak longitudinal strain across the global heart and infarct region was shown for mice 4 wk after MI. (C) AGGF1 protein therapy increased the longitudinal strain ratios in the global myocardial region or the infarcted region. The longitudinal strain ratios across the global heart and infarction region were shown for mice 4 wk after MI. MI mice were treated with recombinant AGGF1 protein (*n* = 15) or control IgG (*n* = 16). Mice with sham operation were used as controls (*n* = 13). Underlying data are shown in S1 Data.
(TIF)

S10 Fig Morphometric analysis of cardiac hypertrophy in mice with AGGF1 treatment and IgG treatment 28 d after MI. (A) Effects of AGGF1 protein therapy on the ratio of heart weight over tibia length. An MI-induced increase in the ratio of heart weight over tibia length was inhibited by AGGF1 protein therapy compared to the treatment with control IgG. (B) Effects of AGGF1 protein therapy on the ratio of lung weight over tibia length. An MI-induced increase in the ratio of lung weight to tibia length was inhibited by AGGF1 protein therapy compared to the treatment with control IgG. (C) Effects of AGGF1 protein therapy on the ratio of heart weight over body weight. An MI-induced increase in the ratio of heart weight to body weight was inhibited by AGGF1 protein therapy compared to the treatment with control IgG. (D) Effects of AGGF1 protein therapy on the ratio of lung weight over body weight. An MI-induced increase in the ratio of lung weight to body weight was inhibited by AGGF1 protein therapy compared to the treatment with control IgG. MI mice were treated with recombinant AGGF1 protein (*n* = 15) or control IgG (*n* = 16). Mice with sham operation were used as controls (*n* = 13). Underlying data are shown in S1 Data.
(TIF)

S11 Fig AGGF1 reduces cardiac necrosis induced by MI and increases α-SMA positive vessels. (A) Hematoxylin and eosin staining of myocardial sections 28 d after LAD ligation (*n* = 5/group). (B) Immunostaining with an anti-α-SMA antibody of myocardial sections 28 d after LAD ligation (*n* = 5/group). Scale bar = 50 μm.
(TIF)

S12 Fig Positive control for cardiac apoptosis. DNase I treatment in cross-sections of mouse hearts was used as a positive control in TUNEL staining. Scale bar = 50 μm.
(TIF)

S13 Fig AGGF1 protein therapy reduces infarct sizes and increases LA wall thickness. (A) Representative images from Masson trichrome staining of cross-sections in the infarct area of hearts 4 wk after MI surgery. MI mice were treated with recombinant AGGF1 protein or control IgG. Mice with sham operation were used as controls. AGGF1 protein therapy inhibited anterior wall fibrosis after MI. The images from Masson trichrome-stained sections in the area of infarction 4 wk after MI surgery were quantified and the data are shown in (B) and (C). Scale bar = 1 mm. (B) AGGF1 protein therapy significantly reduced fibrotic infarct sizes after MI compared to the treatment with control IgG. The fibrotic size measurement was a percentage of the fibrotic area over the LV circumference (*n* = 5/group). (C) AGGF1 protein therapy significantly increased LV wall thickness after MI compared to the treatment with control IgG (*n* = 5/group). MI mice were treated with recombinant AGGF1 protein (*n* = 5) or control IgG (*n* = 5). Mice with sham operation were used as controls (*n* = 5). Underlying data are shown in S1 Data.
(TIF)

S14 Fig Therapeutic benefit of AGGF1 protein was blocked by autophagy inhibitor bafilomycin A1. Representative M-mode echocardiograms are shown for mice 28 d after MI with bafilomycin A1 (Baf) or control vehicle pretreatment followed by treatment with AGGF1 protein or control IgG.
(TIF)

S15 Fig Myocardial injection of AAV9-CMV-Cre viruses eliminated expression of Atg5 in *Atg5*^*flox/flox*^ Mice and blocked autophagy. Western blot analysis showed that AAV-CMV-Cre injection knocked Atg5 expression out and decreased autophagy (LC3-II) in the hearts of *Atg5*^*flox/flox*^ mice.
(TIF)

S16 Fig Atg14L siRNA inhibited autophagy induced by AGGF1. The number of DFCP1-positive puncta was increased by AGGF1 treatment in HUVECs. Knockdown of *Atg14L* using siRNA significantly decreased the number of DFCP1 puncta and blocked the effect of AGGF1. Scale bar = 10 μm.
(TIF)

S1 Table Longitudinal strain and longitudinal strain ratios for different treatment groups after MI. (XLSX)

## Abbreviations

AGGF1: Angiogenic Factor With G-Patch And FHA Domains
AMBRA1: Autophagy And Beclin 1 Regulator 1
Baf: Bafilomycin A1
CABG: Coronary Artery Bypass Surgery
CAD: Coronary Artery Disease
CQ: Chloroquine
dNTP: deoxyribose nucleoside triphosphate
ECs: Endothelial Cells
ER: Endoplasmic Reticulum
FGF2: Fibroblast Growth Factor 2
GFP: Green Fluorescent Protein
HPF: High-Power Field
HUVECs: Human Umbilical Vein Endothelial Cells
HW: Heart Weight
IgG: Immunoglobin G
JNK: c-Jun N-terminal protein kinase
KO: knockout
KTS: Klippel–Trénaunay syndrome
LAD: Left Anterior Descending
LC3: Light Chain 3
LV: Left Ventricular
LVEDD: Left Ventricular End-Diastolic Diameter
LVEF: Left Ventricular Ejection Fraction
LVESD: Left Ventricular End-Systolic Diameter
LVFS: Left Ventricular Fraction Shortening
MI: Myocardial Infarction
PARP: Poly (ADP-ribose) polymerase
PCI: Percutaneous Coronary Intervention
RT: Reverse transcription
siRNA: small interfering RNA
SNARE: Soluble N-ethylmaleimide-sensitive factor attachment protein receptor
TL: Tibia Length
ULK1: Unc-51 Like Autophagy Activating Kinase 1
VEGF: vascular endothelial growth factors
