# Supplementary figures and images for: Angiogenic Factor AGGF1 Activates Autophagy with an Essential Role in Therapeutic Angiogenesis for Heart Disease
Source: PLoS Biol. 2016 Aug 11;14(8):e1002529. doi: 10.1371/journal.pbio.1002529 (PMC4981375; doi:10.1371/journal.pbio.1002529)

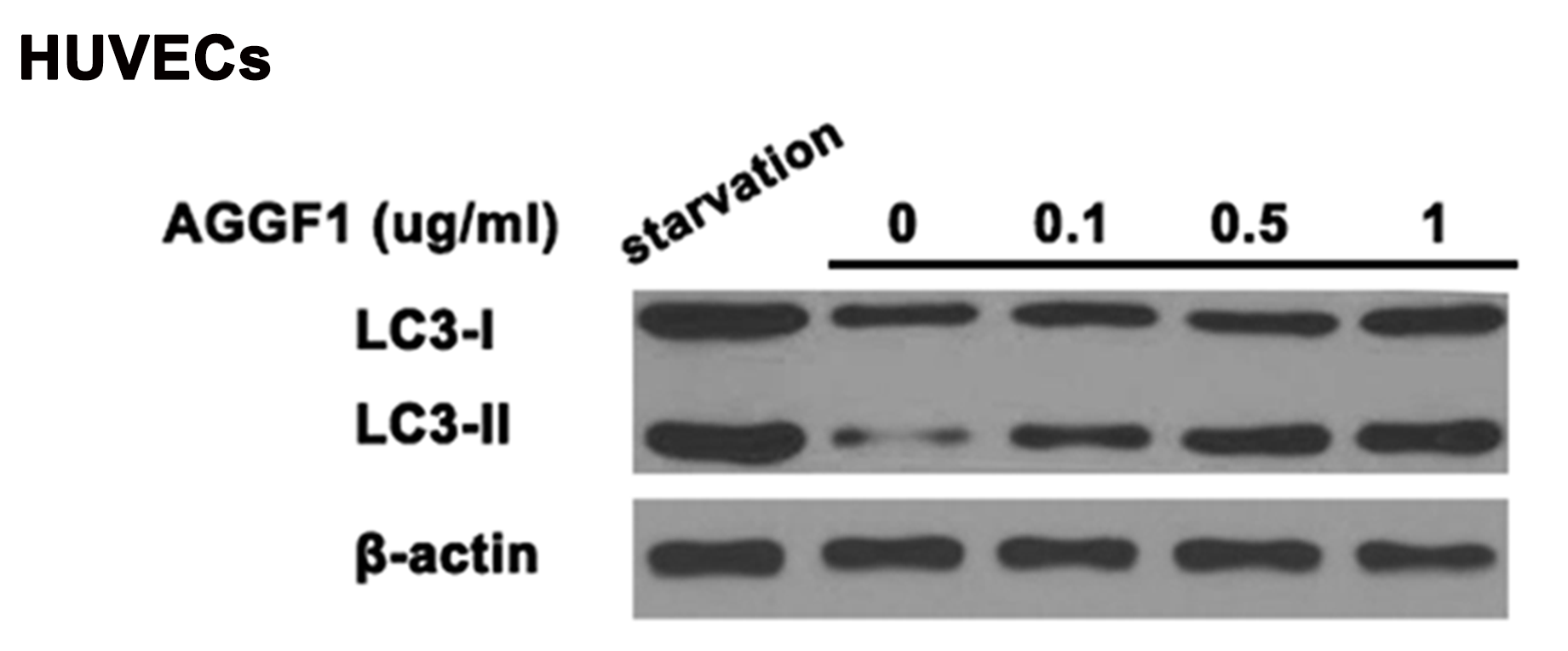

Supplement: S1 Fig — Western blot analysis showed that administration of AGGF1 at different concentrations induced autophagy (increased LC3-II/β-actin and LC3-II/I ratios; decreased p62 expression). Serum starvation was used as positive control for autophagy activation. (TIF) [file pbio.1002529.s002.tif]

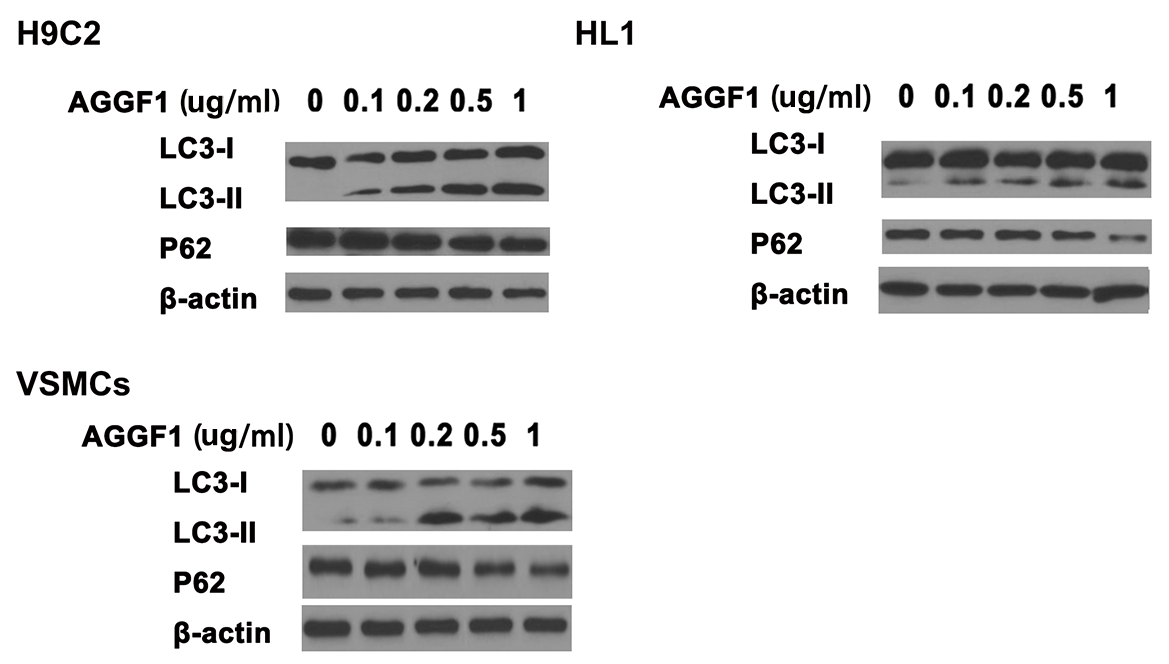

Supplement: S2 Fig — (TIF) [file pbio.1002529.s003.tif]

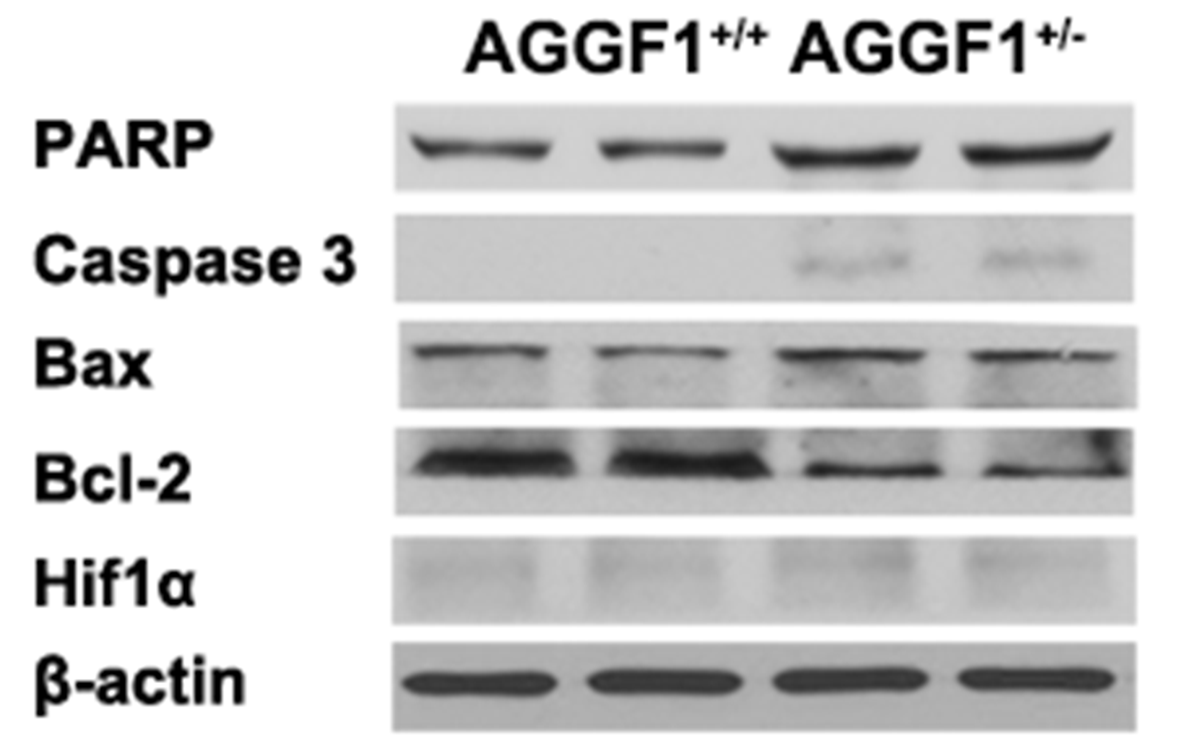

Supplement: S3 Fig — Western blot analysis with apoptosis markers showed that the apoptosis pathway was increased in Aggf1+/- hearts compared to wild-type samples. (TIF) [file pbio.1002529.s004.tif]

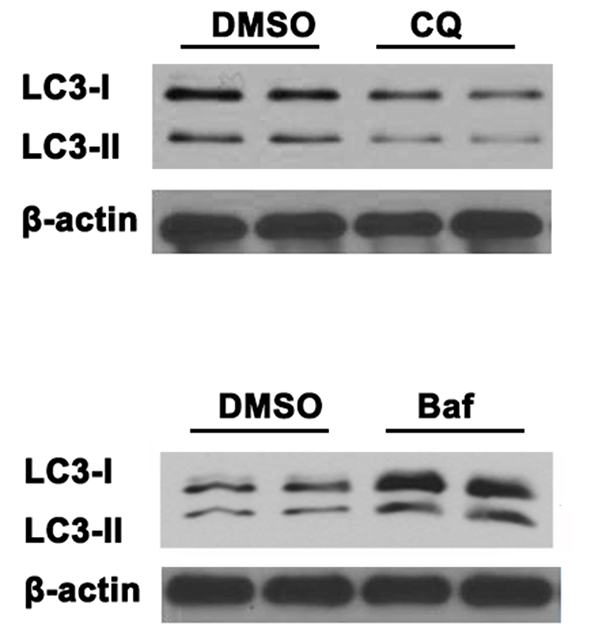

Supplement: S4 Fig — Western blot analysis showed that autophagy inhibitors CQ and Baf inhibited autophagy. Note that Baf inhibits the fusion of autophagosomes with lysosomes and thus inhibits autophagy although LC3-II expression is increased. (TIF) [file pbio.1002529.s005.tif]

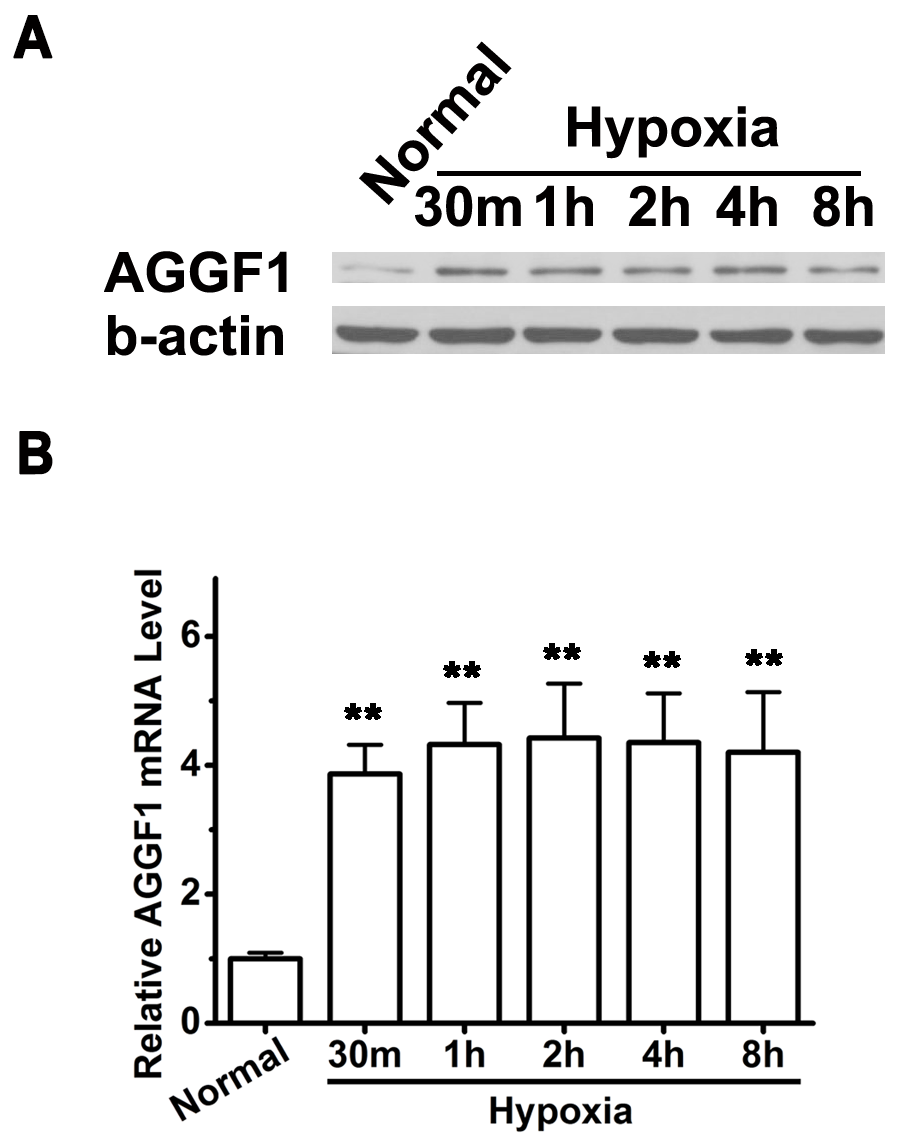

Supplement: S6 Fig — (A) Western blot analysis to measure the AGGF1 protein expression levels in HUVECs under hypoxia with 1% O2 for different time points. The data were from experiments independently repeated at least four times. (B) Quantitative real-time RT-PCR analysis to measure the expression levels of AGGF1 mRNA in HUVECs under hypoxia with 1% O2 for different time points. The data were from experiments independently repeated at least four times. Underlying data are shown in S1 Data. (TIF) [file pbio.1002529.s007.tif]

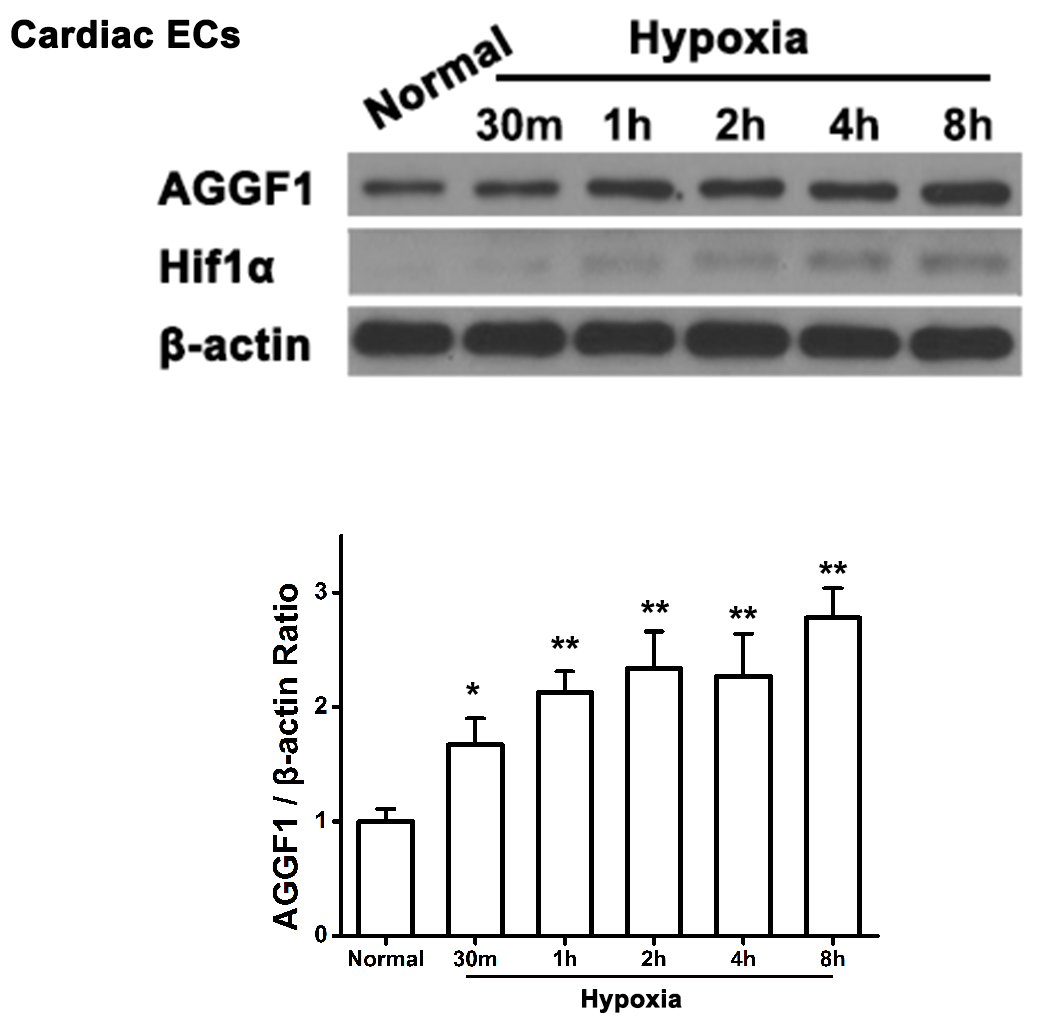

Supplement: S7 Fig — Western blot analysis was used to measure the AGGF1 protein expression levels in ECs isolated from mouse hearts under hypoxia with 1% O2 at different time points. The images were quantified and plotted below. The data were from experiments independently repeated at least three times. Underlying data are shown in S1 Data. (TIF) [file pbio.1002529.s008.tif]

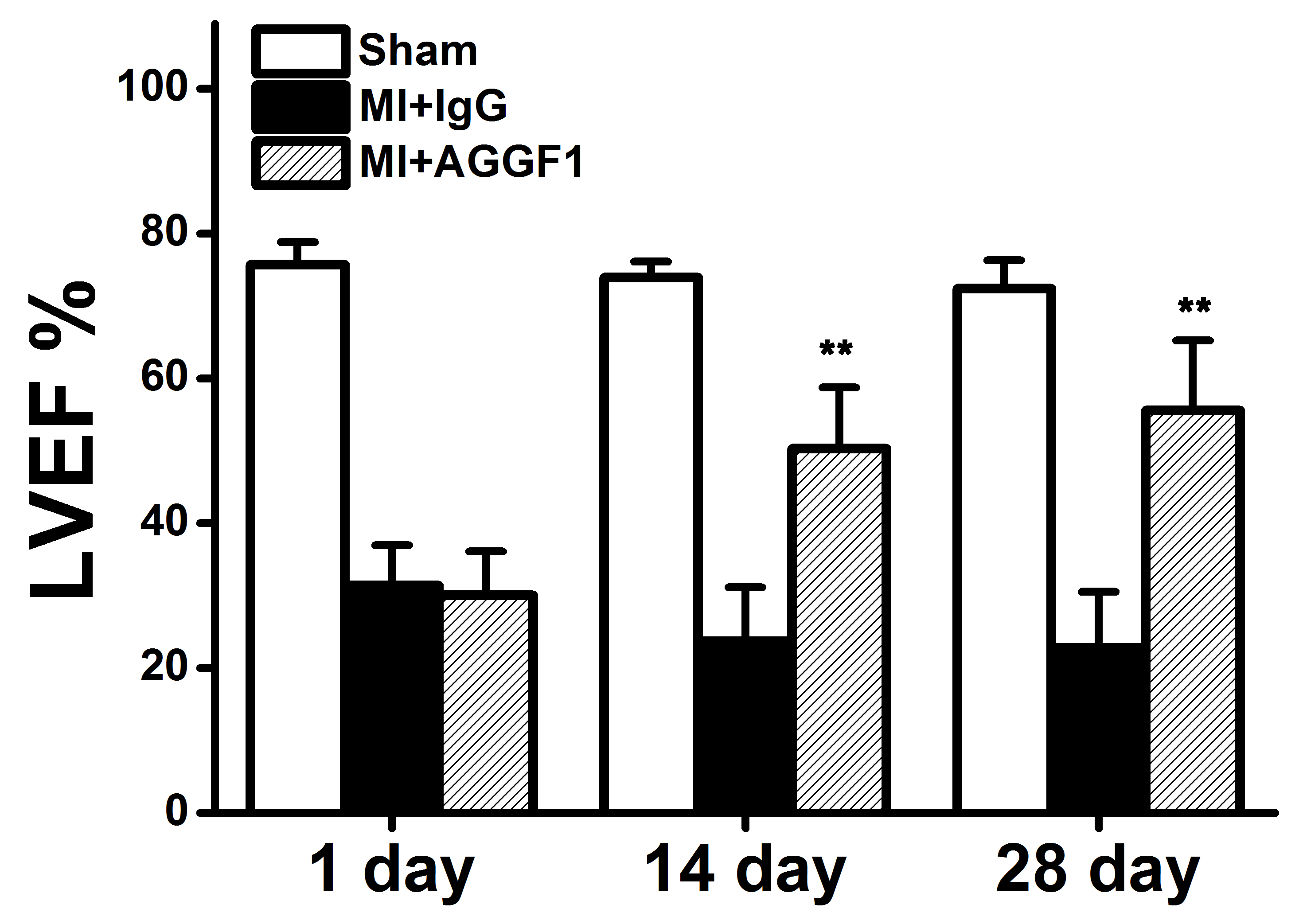

Supplement: S8 Fig — C57BL/6N mice were assessed for baseline cardiac function with echocardiography prior to surgery and then subjected to LAD ligation. One day after the surgery, each animal was examined with echocardiography to confirm the success of the MI surgery. One week after the surgery, the MI mice received AGGF1 or control IgG twice a week for 2 wk or until death. Mice with sham operation were used as controls. The mice were then studied by Vevo-2100 echocardiography. Underlying data are shown in S1 Data. (TIF) [file pbio.1002529.s009.tif]

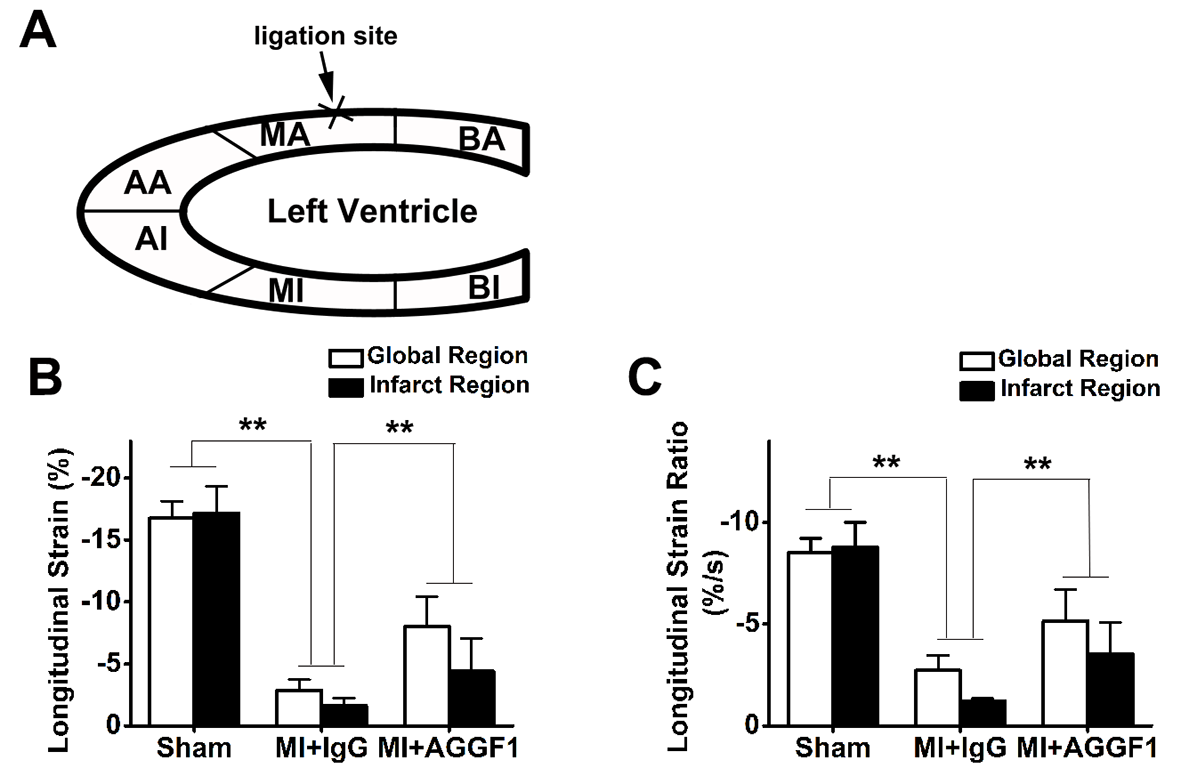

Supplement: S9 Fig — (A) Schematic diagram showing the myocardial regions marked from the paraternal long axis view. AA, apical anterior; MA, mid anterior; BA, basal anterior; AI, apical inferior; MI, mid inferior; BI, basal inferior. (B) AGGF1 protein therapy increased longitudinal strain in the global myocardial region or the infarcted region. Peak longitudinal strain across the global heart and infarct region was shown for mice 4 wk after MI. (C) AGGF1 protein therapy increased the longitudinal strain ratios in the global myocardial region or the infarcted region. The longitudinal strain ratios across the global heart and infarction region were shown for mice 4 wk after MI. MI mice were treated with recombinant AGGF1 protein (n = 15) or control IgG (n = 16). Mice with sham operation were used as controls (n = 13). Underlying data are shown in S1 Data. (TIF) [file pbio.1002529.s010.tif]

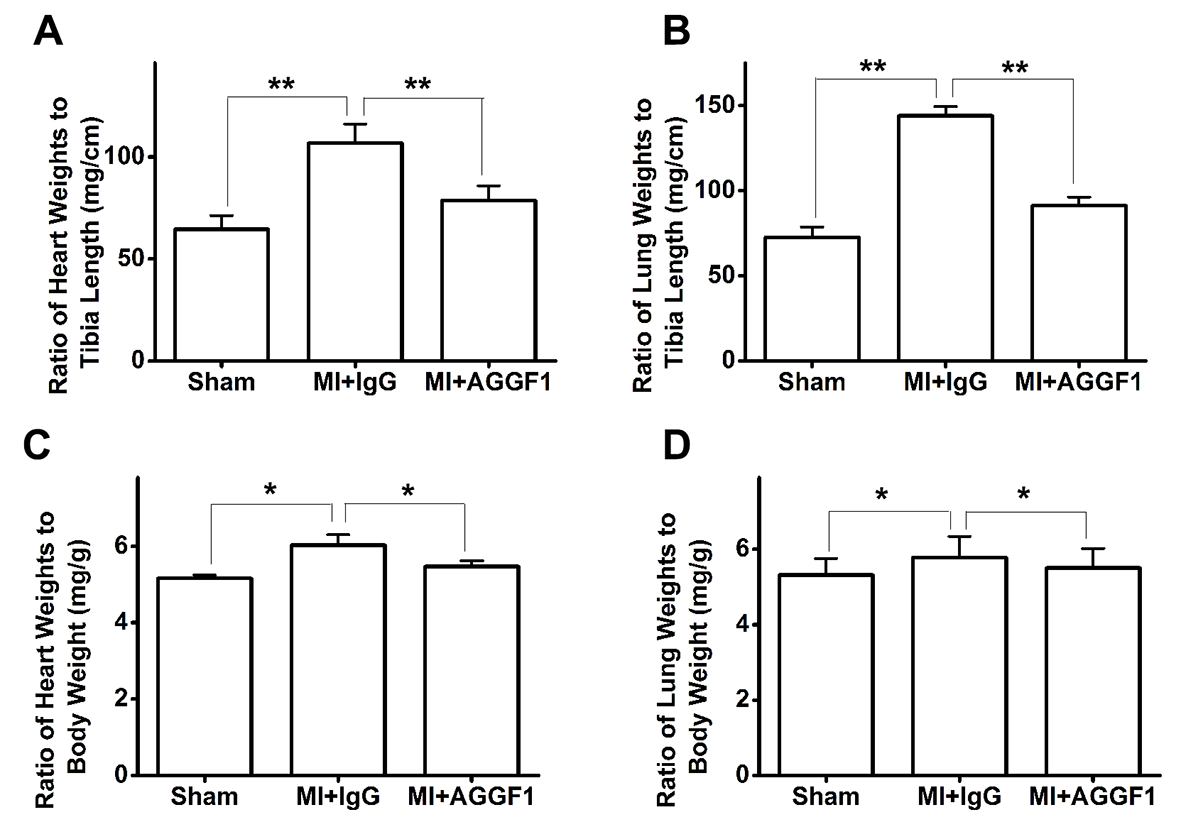

Supplement: S10 Fig — (A) Effects of AGGF1 protein therapy on the ratio of heart weight over tibia length. An MI-induced increase in the ratio of heart weight over tibia length was inhibited by AGGF1 protein therapy compared to the treatment with control IgG. (B) Effects of AGGF1 protein therapy on the ratio of lung weight over tibia length. An MI-induced increase in the ratio of lung weight to tibia length was inhibited by AGGF1 protein therapy compared to the treatment with control IgG. (C) Effects of AGGF1 protein therapy on the ratio of heart weight over body weight. An MI-induced increase in the ratio of heart weight to body weight was inhibited by AGGF1 protein therapy compared to the treatment with control IgG. (D) Effects of AGGF1 protein therapy on the ratio of lung weight over body weight. An MI-induced increase in the ratio of lung weight to body weight was inhibited by AGGF1 protein therapy compared to the treatment with control IgG. MI mice were treated with recombinant AGGF1 protein (n = 15) or control IgG (n = 16). Mice with sham operation were used as controls (n = 13). Underlying data are shown in S1 Data. (TIF) [file pbio.1002529.s011.tif]

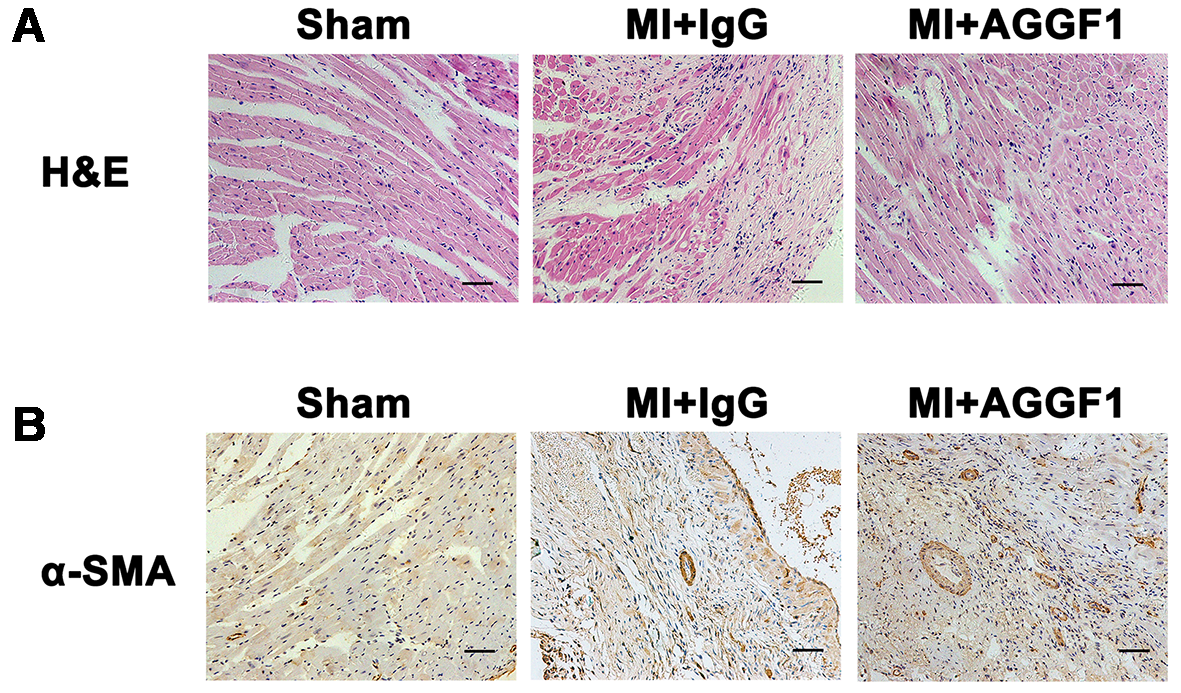

Supplement: S11 Fig — (A) Hematoxylin and eosin staining of myocardial sections 28 d after LAD ligation (n = 5/group). (B) Immunostaining with an anti-α-SMA antibody of myocardial sections 28 d after LAD ligation (n = 5/group). Scale bar = 50 μm. (TIF) [file pbio.1002529.s012.tif]

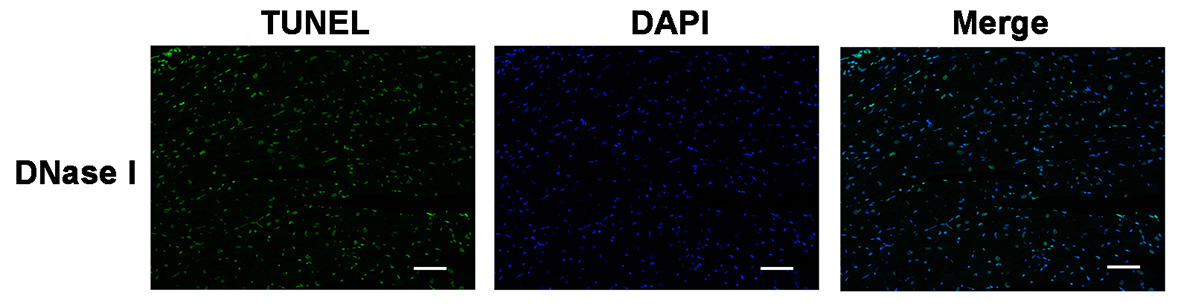

Supplement: S12 Fig — DNase I treatment in cross-sections of mouse hearts was used as a positive control in TUNEL staining. Scale bar = 50 μm. (TIF) [file pbio.1002529.s013.tif]

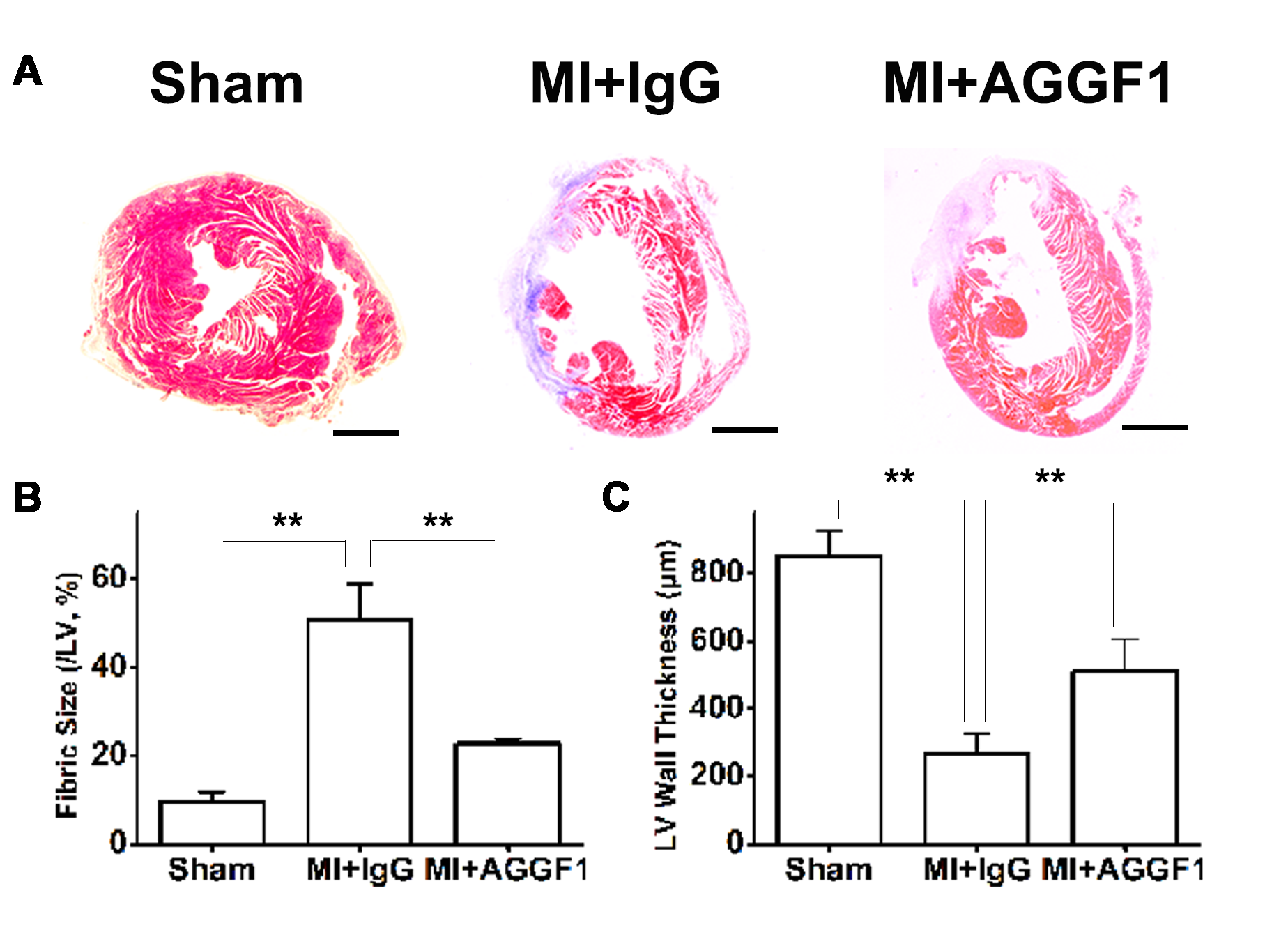

Supplement: S13 Fig — (A) Representative images from Masson trichrome staining of cross-sections in the infarct area of hearts 4 wk after MI surgery. MI mice were treated with recombinant AGGF1 protein or control IgG. Mice with sham operation were used as controls. AGGF1 protein therapy inhibited anterior wall fibrosis after MI. The images from Masson trichrome-stained sections in the area of infarction 4 wk after MI surgery were quantified and the data are shown in (B) and (C). Scale bar = 1 mm. (B) AGGF1 protein therapy significantly reduced fibrotic infarct sizes after MI compared to the treatment with control IgG. The fibrotic size measurement was a percentage of the fibrotic area over the LV circumference (n = 5/group). (C) AGGF1 protein therapy significantly increased LV wall thickness after MI compared to the treatment with control IgG (n = 5/group). MI mice were treated with recombinant AGGF1 protein (n = 5) or control IgG (n = 5). Mice with sham operation were used as controls (n = 5). Underlying data are shown in S1 Data. (TIF) [file pbio.1002529.s014.tif]

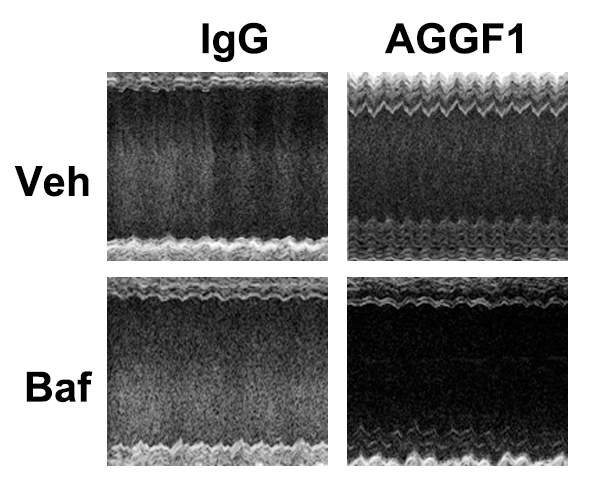

Supplement: S14 Fig — Representative M-mode echocardiograms are shown for mice 28 d after MI with bafilomycin A1 (Baf) or control vehicle pretreatment followed by treatment with AGGF1 protein or control IgG. (TIF) [file pbio.1002529.s015.tif]

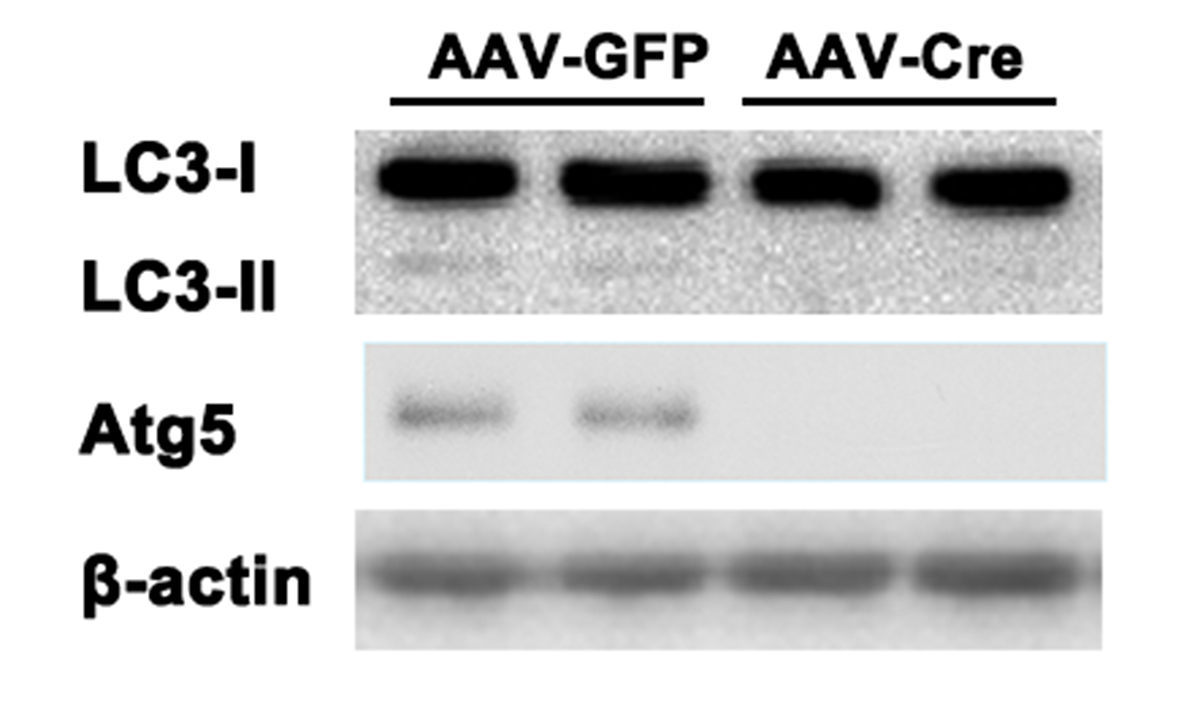

Supplement: S15 Fig — Western blot analysis showed that AAV-CMV-Cre injection knocked Atg5 expression out and decreased autophagy (LC3-II) in the hearts of Atg5flox/flox mice. (TIF) [file pbio.1002529.s016.tif]

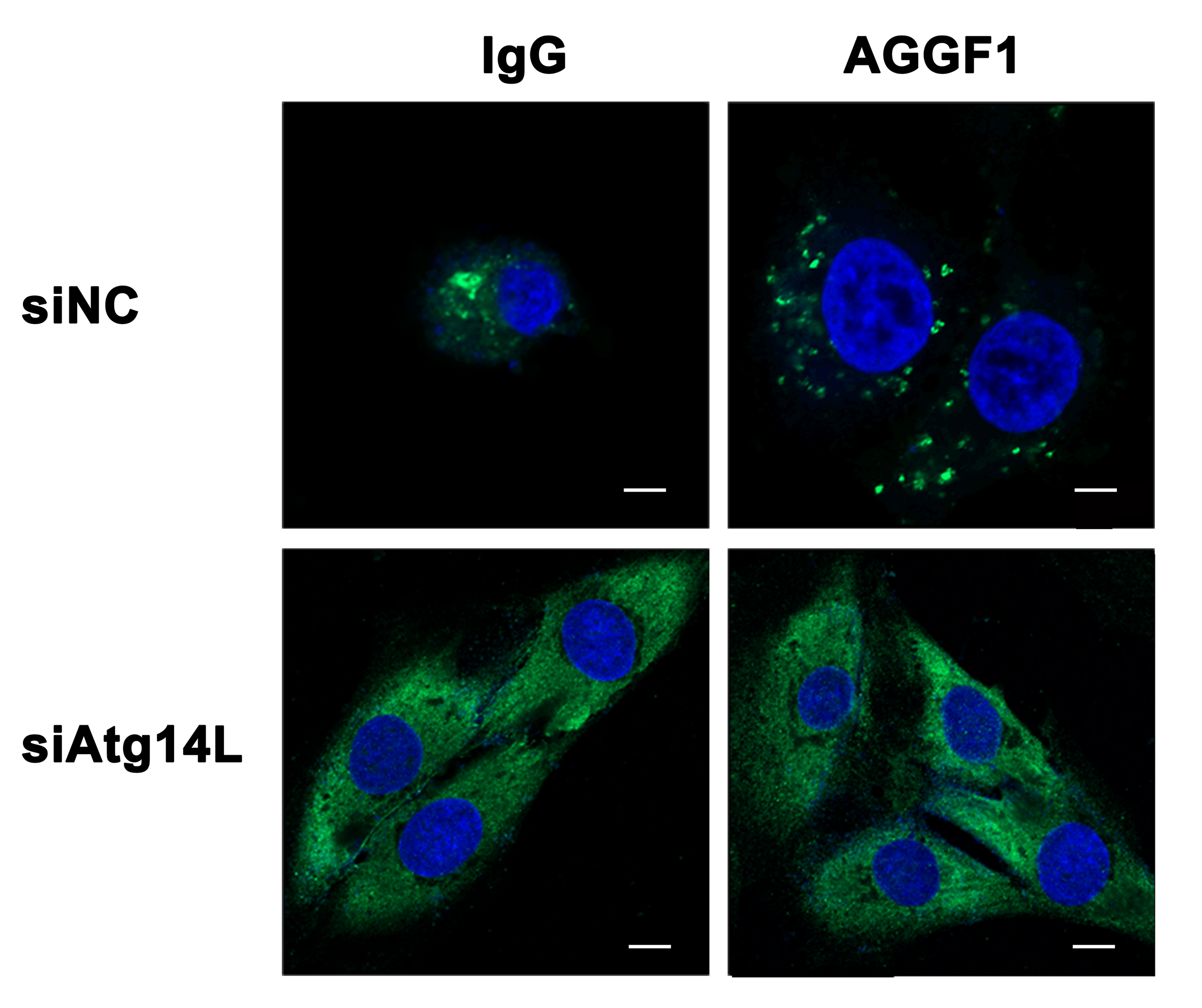

Supplement: S16 Fig — The number of DFCP1-positive puncta was increased by AGGF1 treatment in HUVECs. Knockdown of Atg14L using siRNA significantly decreased the number of DFCP1 puncta and blocked the effect of AGGF1. Scale bar = 10 μm. (TIF) [file pbio.1002529.s017.tif]
